# Integrating genome and RNA sequencing to enhance diagnostic precision in cerebral palsy

**DOI:** 10.1186/s12887-026-06861-z

**Published:** 2026-04-14

**Authors:** Liuyang Zhang, Yiran Xu, Yanqiu Liu, Yongyi Zou, Hui Xiao, Lingling Zhang, Dengna Zhu, Yanan Wu, Xiaoli Zhang, Mirigul Maymaytiniyazi, Bicheng Yang, Changlian Zhu, Tingting Huang

**Affiliations:** 1 Center of Medical Genetics, Jiangxi Maternal and Child Health Hospital, Nanchang, China; 2 The First Affiliated Hospital, Jiangxi Medical College, Nanchang University, Nanchang, China; 3 Henan Key Laboratory of Child Brain Injury and Henan Pediatric Clinical Research Center, The Third Affiliated Hospital and Institute of Neuroscience of Zhengzhou University, Zhengzhou, China; 4 Department of Children Rehabilitation, Third Affiliated Hospital of Zhengzhou University, Zhengzhou, China; 5 Moyu County Maternal and Child Health Hospital, Moyu, China; 6 Center for Brain Repair and Rehabilitation, Institute of Neuroscience and Physiology, University of Gothenburg, Göteborg, Sweden

**Keywords:** Cerebral palsy, Genetic diagnostics, Genome Sequencing, RNA sequencing, Precision medicine

## Abstract

**Background:**

Cerebral palsy (CP), a group of permanent disorders of the development of movement and posture affecting developing fetal or infant brain, shows considerable genetic diversity. Genetic diagnosis is not yet the first-tier diagnostic test for CP. This study aimed to diagnose patients with CP and explore novel diagnostic strategies.

**Methods:**

This study cohort comprised 27 children diagnosed with CP and their asymptomatic parents. Whole-Genome Sequencing and RNA sequencing were employed for genetic diagnostics in these children. The R package Gene Set Variation Analysis was employed to evaluate the pathway enrichment variations between the samples.

**Results:**

Family-based Whole-Genome Sequencing diagnostics identified pathogenic or likely pathogenic variants in 2 children and variants of uncertain significance in 7 children. A total of 216 genes with damaging de novo variant were found, including 3 genes previously associated to CP: *PROC*, *WDR81*, and *SPTBN2*. Additionally, 22 genes contained two or more damaging de novo variants. RNA-seq analysis revealed 5 candidate genes with abnormal expression and 250 candidate events with abnormal alternative splicing. Notably, highly reliable alternative splicing sites were found in *DNMT1* and *VPS13C*. Furthermore, pathway analysis identified aberrant pathways that may help determine the underlying cause. This approach uncovered 3 abnormal pathways related to neurological functions in 2 children and a deleterious de novo variant in the *S1PR4* gene.

**Conclusions:**

These findings supported the application of genetic diagnostics in CP and suggested that a combined approach using genomics and transcriptomics is more comprehensive and reliable. Therefore, we advocated for genetic diagnostics as the first-tier diagnostic test for CP, which not only aids in understanding the etiology but also provides a basis for subsequent treatment strategies.

**Supplementary Information:**

The online version contains supplementary material available at 10.1186/s12887-026-06861-z.

## Background

CP is a group of permanent disorders of the development of movement and posture, attributed to non-progressive disturbances that occurred in the developing fetal or infant brain [1]. The prevalence of CP was estimated as 1.6 per 1000 live births in high-income countries and higher in others [2]. Established risk factors of CP encompass prematurity, intrauterine infections, intra-uterine growth restriction, birth defects, and perinatal asphyxia, of which hypoxia during the birthing process has been previously regarded as a major contributor to the development of CP [3, 4]. Furthermore, more than one-third of children with CP may lack traditional risk factors, while outcomes vary considerably among those with identifiable risk factors [5]. Based on two different large meta-analysis, the application of genetic diagnosis in CP, through whole exome sequencing (WES) or Whole-Genome Sequencing (WGS), achieved a diagnostic rate of 24%~31.1% [6, 7].

However, approximately 69% of children with CP still do not receive a genetic diagnosis after WES or WGS [6, 8]. The limitations of WES and WGS can be compensated by directly detecting changes in RNA abundance by RNA sequencing (RNA-seq), including abnormal gene expression and alternative splicing events [9]. Additionally, the occurrence of diseases is accompanied by pathway abnormalities, and changes in RNA abundance within the relevant pathways can reflect information about the pathway status. To date, fewer studies have been conducted to identify genes associated with CP by tracking abnormally activated or inhibited signaling pathways.

In this study, we performed genetic diagnostics for 27 children with CP via WGS and RNA-seq. We identified pathogenic (P) or likely pathogenic (LP) variants in 2 children and variants of uncertain significance (VUS) in 7 children. We also found 5 genes with aberrant expression and 2 genes with alternative splicing events and reliable splicing variants. Furthermore, we identified 3 aberrant pathways in 2 children. Our findings suggest that a combined approach using genomics and transcriptomics offers more comprehensive opportunities for diagnosing children with CP.

## Methods

### Study cohort

Initially, 27 children with CP and their asymptomatic parents were recruited via the genetic services of The Third Affiliated Hospital of Zhengzhou University. All patients were clinically assessed and diagnosed by referring physicians along with brain MRI detection for abnormalities. Exclusion criteria at enrollment included: (1) evidence of acute or subacute neurological deterioration; (2) loss of previously acquired developmental milestones; (3) progressive abnormalities on neuroimaging; or (4) specific metabolic abnormalities suggestive of an underlying progressive encephalopathy. Children diagnosed before 2 years of age were followed longitudinally until at least 2 years to confirm a stable, non-progressive motor phenotype compatible with CP. Cases showing evolving developmental regression, progressive neurological deterioration, or a phenotype inconsistent with static encephalopathy were subject to diagnostic reassessment and were not retained in the final CP analysis. Fisher’s exact test was used for comparisons of binary clinical variables, with a two-sided *P* value < 0.05 set as the threshold for statistical significance. All children’s guardians provided written consent for the use of samples in research. This study was approved by the Ethics Committee of Zhengzhou University (201201002) in accordance with the Helsinki Declaration.

### DNA extraction

Genomic DNA (gDNA) was extracted from the whole blood of patients and their parents using the QIAGEN DNeasy Blood and Tissue Kit (Qiagen, Stanford, CA, USA). The integrity was confirmed through 1% agarose gel electrophoresis, and the concentration was measured with the Qubit dsDNA HS Assay Kit (Thermo Fisher Scientific, Waltham, MA, USA), ensuring the gDNA was of high quality without visible signs of degradation.

### DNA library construction and sequencing

The gDNA of samples were constructed into libraries using a modified version of the method previously described with MGIEasy DNA Library Prep Kit V1 (Beijing Genomics Institute, BGI) [10]. Briefly, gDNA was fragmented, size-selected with magnetic beads, end-repaired to generate blunt ends, and A-tailed. Later the library adaptors were ligated, and the resulting constructs were PCR-amplified and quality-controlled.

Double-stranded libraries were heat-denatured to single strands, circularized, and enzymatically purified to remove residual linear molecules. The single-stranded circles were then amplified by phi29-mediated rolling-circle amplification to yield DNA nanoballs (DNBs) containing ~ 300 copies of each original molecule.

Massively parallel sequencing was performed on the MGISEQ-2000 platform (BGI, Shenzhen, China) with 150-bp paired-end reads at an average with a depth of 40X. Raw data for each sample was at least 120G bp.

### Alignment and variants calling

First, adapter sequences, low-quality reads and reads shorter than 20 base pairs were removed. Next, the cleaned reads were aligned to GRCh37 (hg19) using the BWA v0.7.13 [11]. Following realignment and quality recalibration, SNVs and InDels were identified using GATK v4.4.0.0 [12]. Finally, Variant annotation was conducted using ANNOVAR (https://annovar.openbioinformatics.org/en/latest/) with the RefSeq RefGene database [13].

### Genetic analysis in genes previously associated to CP

In this study, we systematically reviewed the literature and databases such as OMIM [14], Human Phenotype Ontology, and PubMed to summarize 328 susceptibility genes associated with CP and identified genetic loci within these genes previously associated to CP (Supplementary Table S1). All the variants underwent pathogenicity classification in accordance with the Standards and Guidelines established by the American College of Medical Genetics (ACMG) [15]. Accordingly, variants within genes previously associated to CP were categorized into five predefined classes: P, LP, VUS, likely benign, and benign. Candidate variants in genes previously associated to CP were identified based on the following criteria: (a) functional variants, including missense, frameshift, stop gain, stop loss, start loss, and splice; (b) a minor allele frequency of less than 0.05% in 1000 genomes [16], ChinaMAP [17], ExAC [18], or gnomAD [19]; (c) meeting co-segregation analysis within the pedigree; (d) meeting phenotypic correlation with suspected disease; and (e) meeting other pathogenicity criteria outlined in the ACMG guideline. The evaluation was supplemented by incorporating clinical information from the ClinVar [20] database, alternative splicing results predicted using SpliceAI [21], and predictive results of SIFT [22], CADD [23], and PolyPhen [24] using ANNOVAR [13].

### Identification of damaging de novo variants

Variants were classified as de novo if they were absent in either parent. Damaging de novo variants were identified using a custom R script with the following criteria: (a) the proband was heterozygous or homozygous, while both parents were wild-type homozygotes; (b) the CADD phred score was greater than 20; (c) the SIFT result was Deleterious; (d) the PolyPhen-2 result was probably damaging or possibly damaging. We used a self-developed R script to tally de novo variants and gene counts. The ggplot2 v3.5.1 package was employed for visualization. De novo genes GO and KEGG enrichment analyses were performed using the R packages clusterProfiler v4.16.0 [25] and org.Hs.eg.db, with visualization handled by enrichplot. The heatmap of related pathways was constructed using the R package named pathview v1.48.0 in conjunction with transcriptomic data.

### RNA-seq and analysis

Total RNA was extracted from blood samples of 27 children using the MGIEasy Magnetic Beads Virus RNA Extraction Kit (MGI Tech Co., Ltd., Shenzhen, China) following the manufacturer’s instructions. The quality and quantity of the total RNA were assessed using a Fragment Analyzer and an Agilent 2100 Bioanalyzer (Agilent, CA, USA). Poly (A) mRNA was purified using oligo (dT)-attached magnetic beads. Library construction was performed using the Optimal Dual-mode mRNA Library Prep Kit (BGI-Shenzhen, China). After fragmentation, cDNA was synthesized in two steps: first-strand synthesis primed by random hexamers, then second-strand synthesis. The blunt-ended double-stranded cDNA underwent end repair, after which a single ‘A’ nucleotide was added to the 3’ ends of the blunt fragments through A-tailing. Adaptors were then ligated to the cDNA. After PCR amplification and quality control, single-stranded libraries were denatured and circularized, then amplified into DNBs, carrying over 300 copies each. These DNBs were patterned onto a nanoarray, and sequenced with 150-bp paired-end reads on the MGISEQ 2000 platform (BGI-Shenzhen, China).

The resulting RNA-seq reads were aligned to the hg19 genome assembly using STAR v2.7.10 [26]. Expression levels were quantified via RSEM v1.2.8 [27]. Given the involvement of hundreds of genes in CP, the hypothesis of significant genetic heterogeneity among the pathogenic factors in the 27 probands of this study is tenable. According to this hypothesis, we used the remaining probands as a control group to detect aberrant splicing events.

Furthermore, the LeafCutter software was applied to detect aberrant splicing events [28]. We utilized a custom R script to filter candidate genes with alternative splicing events based on the following criteria: (a) an adjusted *P* value < 0.05; (b) genes previously associated to CP, genes with disease phenotypes related to CP or candidate genes with damaging de novo variants.

### Identification of aberrant pathways

The R package Gene Set Variation Analysis (GSVA v2.2.0) was employed to transform the gene expression matrix into a GSVA score matrix [29], allowing for evaluating the pathway enrichment variations between the samples. The gene sets sourced from the MSigDB database (c2.all.v2023.2.Hs.symbols.gmt) [30]. Pathways were defined as candidate aberrant pathways if one or more probands exhibited GSVA scores below the first quartile minus 1.5 times the interquartile range, or above the third quartile plus 1.5 times the interquartile range. Visualization of aberrant pathway results was accomplished using a custom R script.

## Results

### Clinical characteristics of the CP patients

**Fig. 1:**
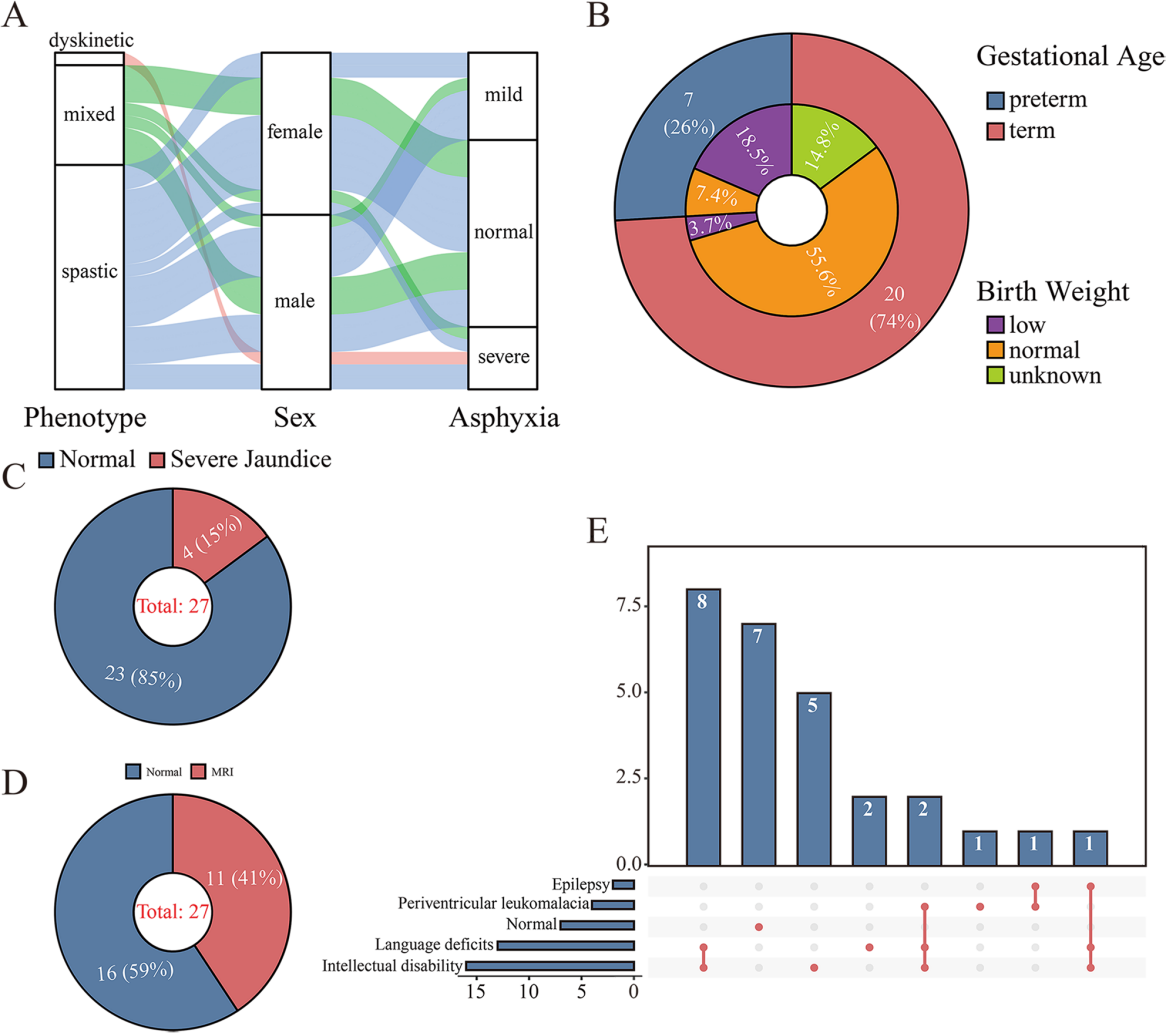
Summary of clinical characteristics of 27 children with CP. **A** Cerebral palsy classification, gender, and history of birth asphyxia. **B** Classifications of gestational age and birth weight. **C** Jaundice history. **D** Magnetic Resonance Imaging (MRI) results. **E** Comorbidities

In this study, we included 27 children with CP with an average age of 3.8 years, along with their biological parents to explore the genetic causes through WGS and RNA-seq. All 27 children underwent comprehensive clinical assessments and then were classified according to data from Surveillance of CP in Europe [31]. Accordingly, 18 were classified as spastic, 1 as dyskinetic, and 8 as mixed. The gender distribution was nearly equal, with 14 males and 13 females. Birth records showed that 12 children had a history of birth asphyxia, with 7 mild and 5 severe, and spastic CP was the most prevalent (Fig. 1A). Gestational age distribution showed that 7 (26%) were preterm, while the rest were term. Regarding birth weight, 6 neonates were classified as low birth weight infants, 17 as normal weight, and 4 were not recorded (Fig. 1B). Fisher’s exact test revealed a statistically significant association between preterm birth and low birth weight (OR = 47.5, 95% CI: 3.55–636.20, *P* = 0.0034). Pie charts in Fig. 1C and D showed that 4 (15%) of the children had severe jaundice and 11 (41%) had MRI results indicating brain abnormalities (including ventriculomegaly, cerebral hypoplasia, periventricular leukomalacia, basal ganglia softening, and cortical sulcal atrophy). Among the 20 patients with comorbidities, 16 had intellectual disability, 13 had language deficits, 4 had periventricular leukomalacia, and 2 had epilepsy (Fig. 1E). Most patients with intellectual disability also present with language deficits.

### Genetic analysis results in genes previously associated to CP

All 27 probands and their parents underwent WGS. Genetic variants potentially implicated in CP were identified in 9 out of 27 families (Table 1). P/LP variants were identified in 2 families, including one family with autosomal recessive (AR) inheritance and another with autosomal dominant (AD)/AR inheritance (Table 1). In detail, c.520 C > T identified in UIG12, was classified as P and located within *PROC* (NM_001375613.1). The *PROC* gene is associated with Thrombophilia 3 due to protein C deficiency, cataloged in OMIM with the identifiers 176,860 and 612,304. In proband UIG008, the c.12dup and c.17del variant, classified as LP, were identified within *ENTPD1* gene (NM_001098175.2). The *ENTPD1* gene is linked to Spastic Paraplegia 64, cataloged in OMIM with the identifier 615,683. VUS variants were found in 7 families, including 2 AD, 2 AR and 2 X-linked (XL), and 1 AD/AR (Table 1). Additionally, a predicted conserved and harmful SNV in the *TENM1* gene was discovered in proband UIG001, despite the absence of a corresponding OMIM disease associated with *TENM1*. This SNV in TENM1 has not been reported nor included in the database. The TEMN1-related mouse models in the Mouse Genome Informatics database indicate that this gene is related to nervous system development.

**Table 1:** Results of ACMG classification in genes previously associated to CP

Sample	Sex	Gene	Protein change	ACMG Classification	OMIM ID	Inheritance
UIG008	female	ENTPD1	p.Lys5GlnfsTer11	LP (PVS1_S+PS2_M+PM2_P)	615,683	AR
			p.Asp6AlafsTer2	LP (PVS1_S+PS2_M+PM2_P)		
UIG12	male	PROC	p.Gln174Ter	P (PVS1+PM2_P+PP5)	176,860	AD, AR
UIG14	female	SPTBN2	p.Glu652Gly	VUS (PS2_M+PM2_P)	600,224	AD, AR
			p.Glu654Gly	VUS (PS2_M+PM2_P)		
UIG031	male	L1CAM	p.Asp1158Gly	VUS (PM2_P+PP3)	303,350	XLR
UIG038	female	DYNC1H1	p.Ile4486Thr	VUS (PS2_M+PM2_P)	614,563	AD
UIG038	female	NTRK1	p.Ala155Thr	VUS (PM2_P+PP3)	256,800	AR
			p.Gly582Asp	VUS (PM2_P+PP3)		
UIG044	male	TUBA1A	p.Ser287Thr	VUS (PS2_M+PM2_P)	611,603	AD
UIG067	male	PDK3	p.Glu17Gly	VUS (PS2_M+PM2_P)	300,905	XLD
UIG144	male	TH	p.Ala317Glu	VUS (PM2_P+PP3)	605,407	AR
			p.Gln150Pro	VUS (PM2_P)		

### Candidate genes with damaging de novo variants

**Fig. 2:**
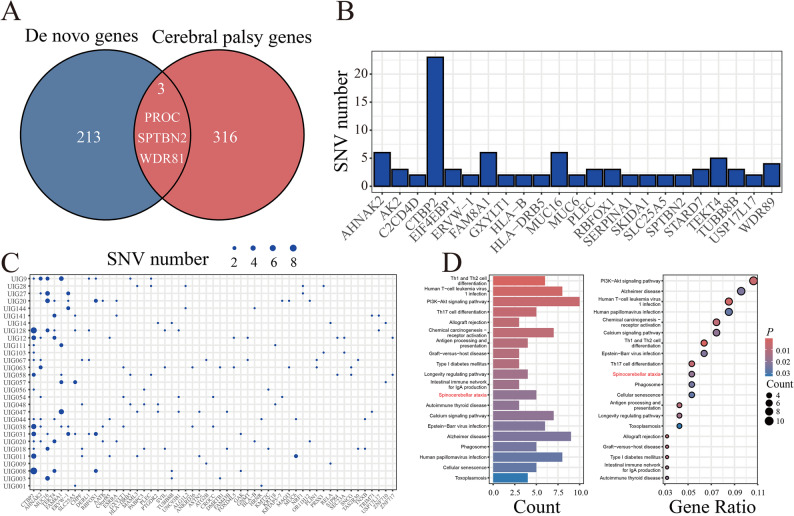
Candidate genes with damaging de novo variants. **A** Venn diagram of candidate genes and genes previously associated to CP. **B** candidate genes had more than two damaging de novo variants. **C** Candidate genes harbored damaging de novo variants in more than two probands. **D** GO and KEGG enrichment results of candidate genes

We identified 283 damaging de novo variants across 216 genes, including three genes previously associated to CP, *PROC*,* WDR81*, and *SPTBN2* (Fig. 2A and Supplementary Table S2). Four damaging de novo variants were identified within these three CP genes, with two occurring in *SPTBN2* (Table 2). Among the 216 candidate genes, 22 had more than two damaging de novo variants (Fig. 2B). *CTBP2* exhibited the greatest number of damaging de novo variants, followed by *AHNAK2*, *MUC16*, and *FAM8A1* (Fig. 2B). Additionally, a subset of candidate genes harbored damaging de novo variants in more than two probands, with *CTBP2* being the most common, followed by *AHNAK2*, *MUC16*, and *TEKT4* (Fig. 2C). Enrichment analysis revealed that these candidate genes are predominantly associated with pathways related to human diseases, including four pathways specifically linked to neurodegenerative diseases, namely Spinocerebellar ataxia (*P* < 0.05), Alzheimer disease (*P* < 0.05), Pathways of neurodegeneration-multiple diseases (*P* < 0.05), and Huntington disease (*P* < 0.05) (Fig. 2D).

**Table 2:** Summary of damaging de novo variants in genes previously associated to CP

Case	Gene	Protein change	Inheritance	SIFT	PolyPhen	CADD	OMIM ID
UIG009	PROC	p.Glu127Gly	AD	0	0.71	32	176,860
UIG14	SPTBN2	p.Glu654Gly	AD	0.01	0.85	26	600,224
UIG14	SPTBN2	p.Glu652Gly	AR	0	0.999	26.9	615,386
UIG011	WDR81	p.Ala1340Val	AR	0	0.987	25.8	610,185, 617,967

### Aberrant alternative splicing events

For alternative splicing analysis, our study have used the remaining probands as a control group for each sample and detected significant aberrant alternative splicing within 250 clusters (adjusted *P* < 0.05) across 28 genes in 12 probands (Supplementary Table S3). Comparison with WGS results revealed five splicing sites in *DNMT1*, *SERPINA1*, *VPS13C*, and *NDUFS2* (Supplementary Table S4). Using spliceAI, we identified reliant donor loss sites in *DNMT1* (DS_DL = 0.93) and *VPS13C* (DS_DL = 0.95), with Delta score exceeding 0.8, indicating a high probability that these variants will alter splicing (Supplementary Table S4). However, these sites were inherited from their asymptomatic parents.

### Aberrant pathways in probands

**Fig. 3:**
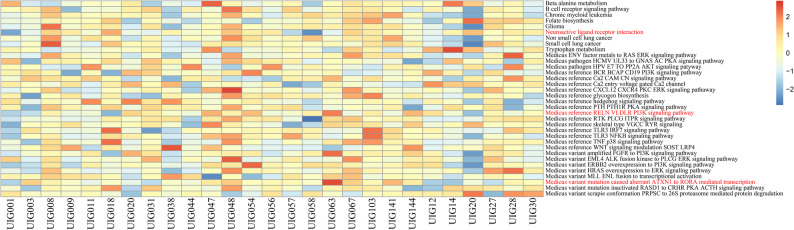
Heatmap of 34 gene sets with at least one proband showing an outlier GSVA score. Heatmap displays GSVA scores, with each column representing a sample and each row a pathway

**Fig. 4:**
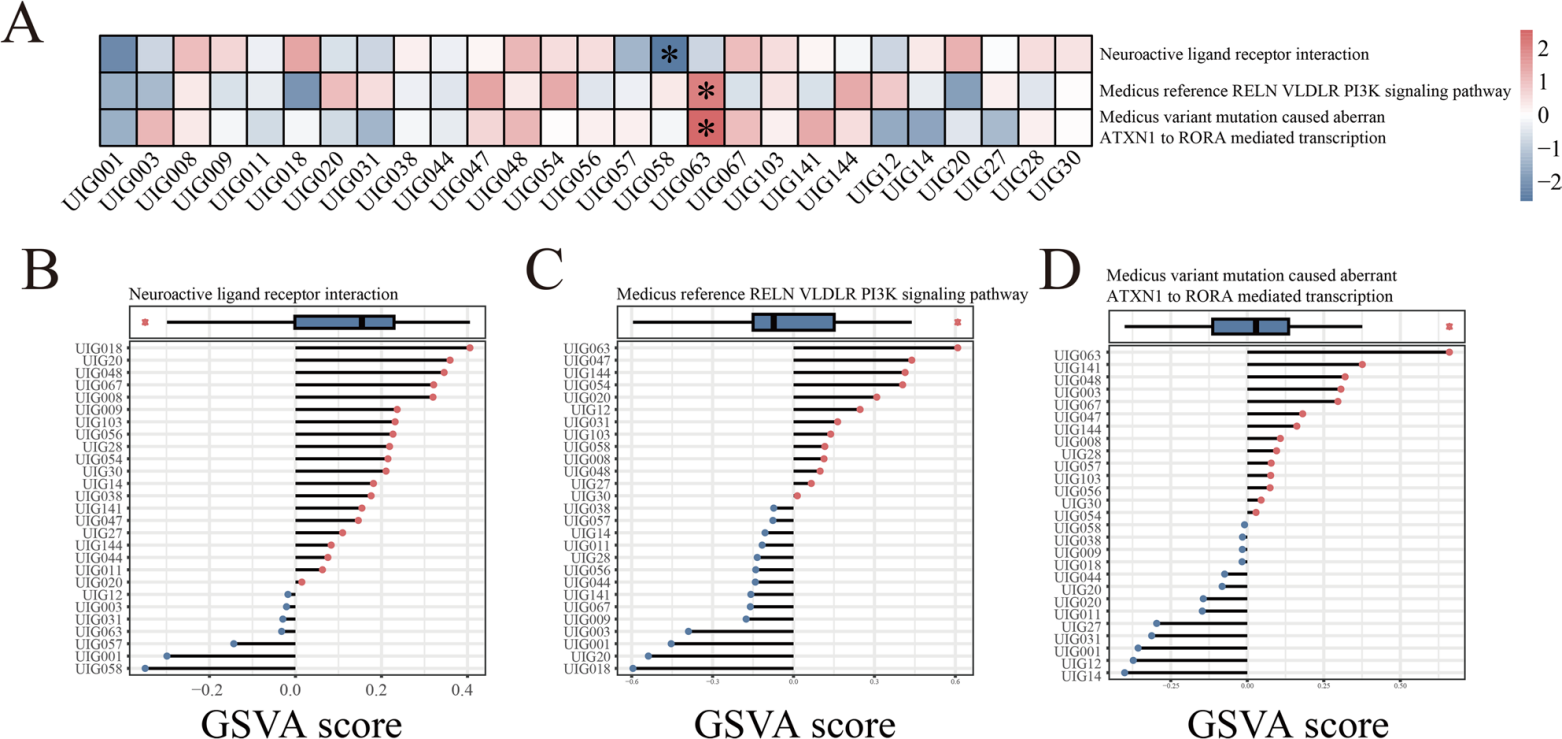
Aberrant pathways in two children with CP. **A** Heatmaps of three pathways related to neurological functions, with asterisks indicating aberrant children. **B**-**D** Box plots and lollipop plots of three abnormal pathways, with red dots representing activation, blue dots representing inhibition, and red stars representing outliers

**Fig. 5:**
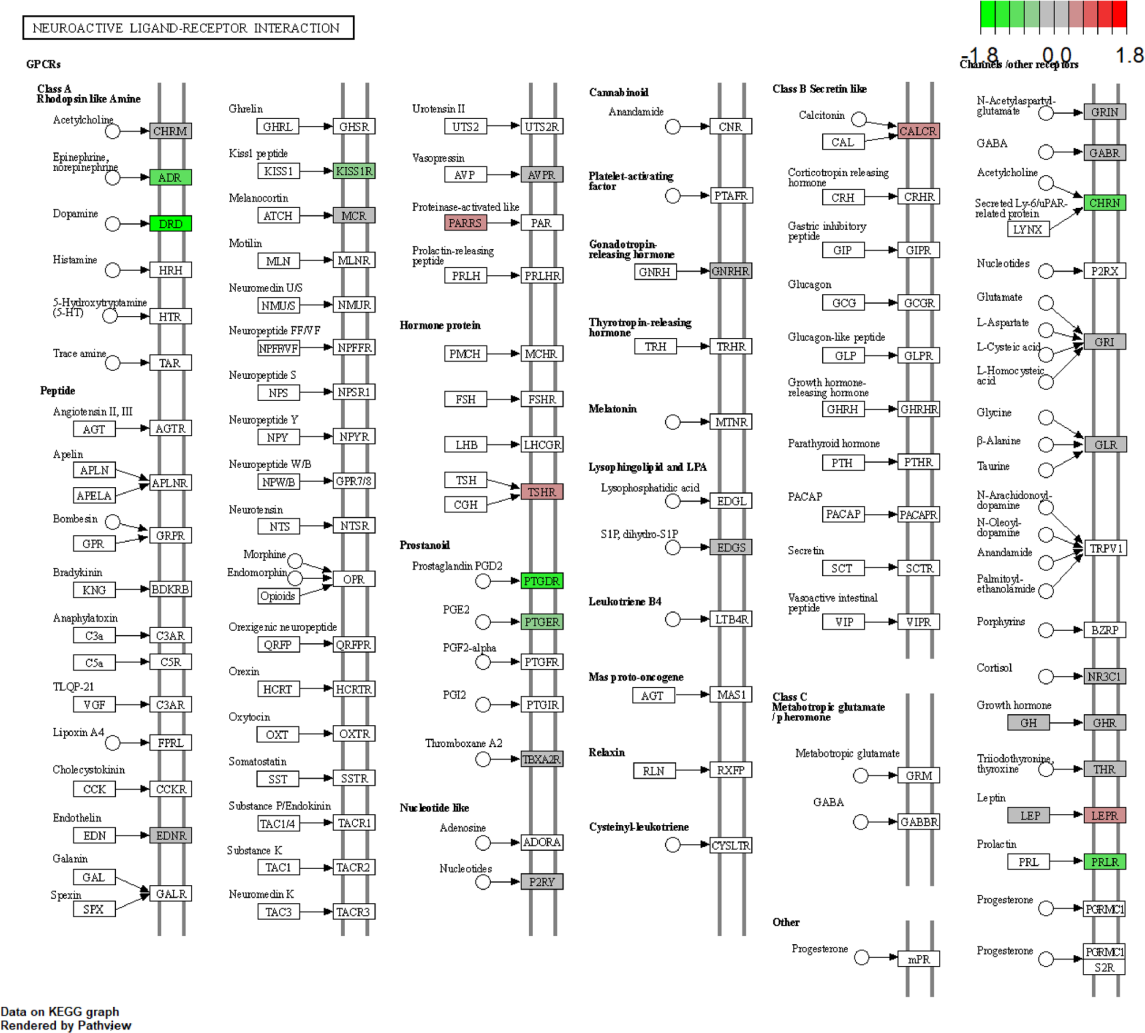
Pathway plot of Neuroactive ligand-receptor interaction pathway in UIG058. Different colors represent the fold changes for each gene in the pathway

The progression of diseases is often accompanied by abnormal activation or inhibition of key cellular signaling pathways. To identify aberrant pathways, we converted the expression matrix into GSVA scores and evaluated the pathway enrichment for each sample. Accordingly, we identified 34 gene sets with at least one proband showing an outlier GSVA score, indicating abnormal activation or repression (Fig. 3). Among these, three gene sets associated with neurological functions were particularly notable: Neuroactive ligand-receptor interaction, RELN-VLDLR-PI3K signaling pathway, and Mutation-caused aberrant ATXN1 to RORA-mediated transcription (Fig. 4A). In proband UIG058, the Neuroactive ligand-receptor interaction pathway showed abnormal inhibition (Fig. 4B). Within this gene set, the *S1PR4* gene harbors a damaging de novo variant (ENST00000246115.5: c.71T > C) in UIG058, despite no significantly different expression compared to other probands (Supplementary Table S2 and Fig. 5). In proband UIG063, the RELN-VLDLR-PI3K signaling pathway and Mutation-caused aberrant ATXN1 to RORA-mediated transcription were abnormally activated (Fig. 4C and D). Both gene sets are classified within the pathway class of Spinocerebellar ataxia.

## Discussion

Although genetic diagnosis is not currently the first-tier diagnostic test for CP, substantial research underscores the importance of WES and WGS in CP diagnosis [6, 32]. This study highlights the utility of these techniques, with experienced clinicians diagnosing two children with variants in the *PROC* and *ENTPD1* genes using stringent ACMG criteria. Moreover, a conserved and deleterious variant in the *TENM1* gene, involved in neurodevelopment, was identified, supporting the use of WGS for CP diagnostic evaluation and revealing its potential in identifying new candidate genes.

Variant in the *PROC* gene, which encodes a vitamin K-dependent plasma glycoprotein can lead to protein C deficiency, a prothrombotic risk factor associated with CP [33]. Previous reports identified compound heterozygous variant in *PROC* in patients with spastic diplegia and spastic quadriplegia, as well as mixed spastic/dyskinetic quadriplegic CP [34, 35]. Similarly, a de novo heterozygous variant in the *PROC* gene was identified in UIG12 in this study [36]. The *ENTPD1* gene encodes ectonucleoside triphosphate diphosphohydrolase 1, with biallelic variants leading to Spastic paraplegia type 64, a recessive hereditary neurological disease [37]. UIG008 exhibited symptoms matching this condition, including low birth weight, delayed intellectual development, severe asphyxiation, and periventricular leukomalacia on MRI [38, 39]. *TENM1*(teneurin transmembrane protein 1, also known as *ODZ3*, or *Ten-m1*), located on the X chromosome, is a member of the teneurin family of transmembrane proteins, which are highly conserved across species and play critical roles in neural development [40]. *TENM1* is predominantly expressed in the developing and adult nervous system, consistent with a role in neurodevelopmental disorders. Multiple research results have confirmed that teneurins play a central role in multiple physiological functions such as synaptic growth, axon guidance, transcriptional regulation, and neuronal migration [41–45]. A hemizygous variant was detected in *TENM1* in UIG001 and is predicted to be deleterious. Notably, a previous whole-exome sequencing study identified a hemizygous *TENM1* variant in a cerebral palsy cohort, also proposing it as a novel candidate CP gene [46]. Consistent with this, the GenCC database lists *TENM1* with limited evidence for CP (X-linked inheritance). Functional constraint metrics indicate that *TENM1* is highly intolerant to loss-of-function variation (pLI = 1.00, LOEUF = 0.19), supporting a potential role in neurodevelopmental disorders.

Additionally, VUSs were identified in seven genes previously associated to CP, including *SPTBN2*,* L1CAM*,* TUBA1A*,* PDK3*, *DYNC1H1*,* NTRK1*, and *TH*. While these VUSs conform to the genetic pattern of CP [46–49], further functional studies, clinical data, and bioinformatics analysis are needed to determine their pathogenicity.

The high genetic heterogeneity of CP is widely acknowledged, with many CP-related genes still unknown [32, 46, 50]. Including parents in trio sequencing helps determine the inheritance pattern of variants and is crucial for identifying Mendelian genetic diseases [51]. This study found 283 damaging de novo variants in 216 genes, including genes previously associated to CP: *PROC*,* WDR81*, and *SPTBN2*. These genes were significantly enriched in neurodegenerative disease pathways. Misdiagnosis of some childhood neurodegenerative diseases as CP, due to symptom similarity [52], underscores the need for genomic tests to clarify diagnoses and inform treatment plans [53, 54]. Trio sequencing enhances diagnostic accuracy and helps identify new genes and loci.

Although genomic sequencing is crucial for diagnosing genetic diseases, it has limitations in some Mendelian disorders. The combination of genomic and transcriptomic data significantly improves diagnostic rate [55]. RNA-seq can diagnose an additional 5% of patients [56]. Given the high genetic heterogeneity, patient samples can serve as controls in genetic diagnosis via RNA-seq, reducing additional control test costs [9]. The alternative splicing analysis identified credible variants in DNMT1 and VPS13C; however, both events were inherited from asymptomatic parents. In the absence of supporting evidence—such as reduced penetrance, tissue-specific expression, or functional validation—these splicing abnormalities cannot be interpreted as pathogenic. Accordingly, they are presented as candidate molecular findings warranting further investigation. Moreover, these observations underscore the need for improved genome annotation, particularly in non-exonic regions, and more advanced interpretation methods to fully leverage transcriptomic data.

To further explore the expression data, a novel approach was used to identify aberrant pathways by converting the expression matrix into a GSVA score matrix. Three aberrant pathways related to spinocerebellar ataxia, were identified in two probands [52]. A damaging de novo variant in the *S1PR4* gene, involved in neuronal activity regulation, was discovered [57], necessitating further investigation.

We acknowledge that family-based WGS and RNA-seq is not yet standard of care and cost is a major barrier. The added value of RNA-seq beyond WGS is most pronounced in cases where DNA‑based testing yields variants of uncertain significance or when a strong clinical suspicion remains despite negative DNA findings. Thus, a staged approach—starting with family-based WGS, followed by RNA-seq in unresolved cases—may be a more cost‑effective implementation strategy.

One limitation of this study is the absence of pathogenic CNVs, which might result from the low prevalence of pathogenic CNVs associated with CP [7]. Another limitation is that the expression profiles were obtained from whole blood samples due to the inaccessibility of CP-related tissues, despite whole blood exhibiting expression similarities with various neural tissues [58]. Due to the small cohort size (*n* = 27), our findings should be considered exploratory. Independent replication in larger, well-phenotyped cohorts is essential to validate these observations.

## Conclusions

Our study identified P/LP variants in genes previously associated to CP including *PROC* and *ENTPD1* with stringent diagnostic criteria. Numerous candidate genes were also identified through de novo variants and transcriptome sequencing data. We pioneered converting expression profiles into pathway enrichment matrices, revealing aberrant pathways and offering new insights for transcriptome-based diagnostics. These findings support the utility of WGS and RNA-seq in diagnosing CP and identifying new causative genes. 

## Supplementary Information

Supplementary Material 1.

Supplementary Material 2.

Supplementary Material 3.

Supplementary Material 4.

Supplementary Material 5.

## Data Availability

The dataset supporting the conclusions of this article is available in the Figshare repository, [https://figshare.com/,doi:10.6084/m9.figshare.29766905].

## Abbreviations

CP: Cerebral palsy
WES: Whole exome sequencing
WGS: Whole-Genome Sequencing
RNA-seq: RNA sequencing
P: Pathogenic
LP: Likely pathogenic
VUS: Variants of uncertain significance
gDNA: Genomic DNA
DNBs: DNA nanoballs
ACMG: American College of Medical Genetics
AR: Autosomal recessive
AD: Autosomal dominant
XL: X-linked
