# Supplementary material for: Integrating genome and RNA sequencing to enhance diagnostic precision in cerebral palsy
Source: BMC Pediatr. 2026 Apr 14;26:484. doi: 10.1186/s12887-026-06861-z (PMC13202834; doi:10.1186/s12887-026-06861-z)
Supplement: Supplementary file 1 — Supplementary Material 1. [file 12887_2026_6861_MOESM1_ESM.docx]

**Supplementary Table S1.** **List of susceptibility genes associated with Cerebral palsy (CP).**

|  | **Gene symbol** |
| --- | --- |
| **Genes associated with CP** | *AARS, ABCD1, ACADM, ACOX1, ACSL4, ADCY5, ADD3, ADGRG1, AFG2B, AIFM1, AIP, ALAD, ALDH18A1, ALDH3A2, ALS2, AMER1, AMPD2, ANG, AP4B1, AP4E1, AP4M1, AP4S1, AP5Z1, APTX, AR, ARG1, ARHGEF10, ARL6IP1, ARNT2, ARSA, ARX, ASPA, ATL1, ATM, ATP1A3, ATP13A2, ATP7A, ATP8A2, ATRX, AUTS2, B4GALNT1, BAG3, BCAP31, BICD2, BRWD3, BSCL2, BTD, C12orf65, C19orf12, CA8, CACNA1A, CACNA1D, CACNB4, CAPN1, CBS, CCT5, CHMP2B, CLCN4, COASY, COL4A1, COQ8A, COX6A1, CP, CPT1C, CTDP1, CTNNB1, CYB5R3, CYP27A1, CYP2U1, CYP7B1, DARS, DCAF17, DCTN1, DDC, DDHD1, DDHD2, DDX3X, DHTKD1, DLG3, DNAJB2, DNASE1L3, DNM2, DNMT1, DOCK6, DSTYK, DYNC1H1, EGR2, ENTPD1, ERBB3, ERLIN1, ERLIN2, ETFDH, ETHE1, EXOSC3, FA2H, FARS2, FAT4, FBLN5, FGD4, FGF14, FIG4, FRMPD4, FTL, FTSJ1, FUS, FXN, F5, GAD1, GALC, GAN, GARS, GBA, GBA2, GBE1, GCDH, GCH1, GDAP1, GDI1, GJA1, GJB1, GJC2, GLA, GLE1, GNAO1, GNB4, GPR101, GRIA3, HACE1, HARS, HCFC1, HESX1, HEXA, HINT1, HK1, HOXD10, HPGD, HPRT1, HSPB1, HSPB3, HSPB8, HSPD1, IBA57, IDS, IDUA, IFIH1, IGHMBP2, IL1RAPL1, INF2, IQSEC2, ITPR1, KANK1, KARS, KCNA1, KCNC3, KDM5C, KIDINS220, KIF1A, KIF1B, KIF1C, KIF4A, KIF5A, KLC2, KLHL15, L1CAM, LAMB1, LAS1L, LITAF, LMNA, LRSAM1, LYST, MAG, MARS, MARS2, MCCC1, MECP2, MED25, MEGF10, MFN2, MID2, MMACHC, MPZ, MTHFR, MTMR2, MTR, MTRR, MTTP, MUT, MYBPC1, MYH14, NDRG1, NEFH, NEFL, NEU1, NEXMIF, NGF, NIPA1, NSRP1, NT5C2, NTRK1, OGT, OPA3, OPTN, PAK3, PANK2, PAX3, PCCA, PCCB, PDHA1, PDHX, PDK3, PEX3, PEX7, PFN1, PGAP1, PHYH, PIP5K1C, PLA2G6, PLEKHG5, PLP1, PMP22, PNPLA6, POLG, PRKCG, PRNP, PROC, PRPH, PRPS1, PRRT2, PRUNE1,* *PRX, PTS, RAB18, RAB39B, RAB3GAP1, RAB3GAP2, RAB7A, REEP1, REEP2, RETREG1, RLIM, RNASEH2B, RNF125, RNF170, RPS6KA3, RTN2, SACS, SBF1, SBF2, SCN2A, SCN4A, SCN8A, SCN9A, SETX, SH3TC2, SIGMAR1, SIL1, SLC12A6, SLC16A2, SLC1A3, SLC25A15, SLC30A10, SLC33A1, SLC5A7, SLC6A3, SLCO2A1, SMARCA4, SMARCB1, SMN1, SMN2, SOD1, SOX10, SPART, SPAST, SPG11, SPG21, SPG7, SPR, SPTBN2, SPTLC1, SPTLC2, STXBP1, SYP, TARDBP, TBC1D20, TBL1XR1, TDP1, TECPR2, TFG, TH, THOC2, TOE1, TREX1, TRIM2, TRPV4, TSPAN7, TTBK2, TTPA, TTR, TUBA1A, TUBB2B, TUBB4A, TWNK, UBA1, UBE3A, UBQLN2, UCHL1, UGT1A1, UNC80, UROD, USP27X, USP8, USP9X, VAMP1, VAPB, VCP, VLDLR, VPS37A, VRK1, WASHC5, WDR45, WDR81, WNK1, YARS, ZC4H2, ZDHHC15, ZFYVE26, ZFYVE27, ZNF711* |
