# Supplementary material for: Integrating genome and RNA sequencing to enhance diagnostic precision in cerebral palsy
Source: BMC Pediatr. 2026 Apr 14;26:484. doi: 10.1186/s12887-026-06861-z (PMC13202834; doi:10.1186/s12887-026-06861-z)
Supplement: Supplementary file 2 — Supplementary Material 2. [file 12887_2026_6861_MOESM2_ESM.docx]

**Supplementary Table S2.** **List of damaging de novo mutations.**

| SampleID | SYMBOL | HGVSc | SIFT | PolyPhen | CADD_phred |
| --- | --- | --- | --- | --- | --- |
| UIG001 | *HRNR* | c.6794A>T | 0.02 | 0.637 | 20.9 |
| UIG001 | *SLC25A5* | c.597G>T | 0 | 0.689 | 24.8 |
| UIG003 | *AK2* | c.668T>C | 0 | 0.931 | 28.5 |
| UIG003 | *AK2* | c.614G>A | 0.02 | 0.905 | 25.4 |
| UIG003 | *AK2* | c.602A>T | 0.01 | 1 | 28.5 |
| UIG003 | *DMRTB1* | c.167T>C | 0 | 0.998 | 28 |
| UIG003 | *GLDC* | c.1202C>T | 0 | 0.663 | 27.8 |
| UIG003 | *TUBB8B* | c.307A>G | 0.01 | 0.89 | 22 |
| UIG003 | *FGF22* | c.463A>G | 0.04 | 0.799 | 23.8 |
| UIG003 | *MUC16* | c.40766A>G | 0.03 | 0.968 | 21.9 |
| UIG003 | *MUC16* | c.40754C>T | 0.04 | 0.964 | 20.4 |
| UIG003 | *MUC16* | c.40753A>C | 0 | 0.964 | 21.9 |
| UIG008 | *TEKT4* | c.1157C>G | 0 | 0.919 | 22.6 |
| UIG008 | *TEKT4* | c.1210A>G | 0.04 | 0.77 | 20.3 |
| UIG008 | *GAK* | c.521T>A | 0 | 0.998 | 28 |
| UIG008 | *CTBP2* | c.2144C>A | 0 | 1 | 27.6 |
| UIG008 | *CTBP2* | c.2143G>A | 0.02 | 0.982 | 26.6 |
| UIG008 | *CTBP2* | c.2141G>T | 0.01 | 0.975 | 26.2 |
| UIG008 | *CTBP2* | c.2125C>G | 0.01 | 0.92 | 29.1 |
| UIG008 | *CTBP2* | c.2113G>A | 0.03 | 0.68 | 26.5 |
| UIG008 | *CTBP2* | c.2072G>C | 0.01 | 0.964 | 32 |
| UIG008 | *CTBP2* | c.2069C>G | 0.01 | 0.991 | 29.8 |
| UIG008 | *CTBP2* | c.2059C>T | 0.01 | 0.997 | 28.4 |
| UIG008 | *PDIA3* | c.121G>T | 0 | 0.903 | 31 |
| UIG008 | *SORD* | c.286C>T | 0 | 1 | 25 |
| UIG008 | *RBFOX1* | c.112G>T | 0 | 0.991 | 27.8 |
| UIG008 | *RBFOX1* | c.113G>T | 0.02 | 0.88 | 25.6 |
| UIG008 | *TUBB8B* | c.569A>G | 0 | 0.943 | 21.5 |
| UIG009 | *STARD7* | c.110G>A | 0.02 | 0.635 | 27.4 |
| UIG009 | *STARD7* | c.107G>C | 0 | 0.992 | 32 |
| UIG009 | *STARD7* | c.104T>C | 0.01 | 0.503 | 28.6 |
| UIG009 | *PROC* | c.380A>G | 0 | 0.71 | 32 |
| UIG009 | *AGAP6* | c.1479C>G | 0 | 0.999 | 20.5 |
| UIG009 | *TMEM132A* | c.1499A>G | 0 | 0.971 | 26.6 |
| UIG009 | *RELA* | c.836A>G | 0 | 0.984 | 29.7 |
| UIG009 | *CDH24* | c.2141A>G | 0 | 0.496 | 27.7 |
| UIG009 | *ATG2B* | c.98A>G | 0 | 0.829 | 32 |
| UIG009 | *CACNA1H* | c.374A>T | 0 | 0.946 | 29.6 |
| UIG009 | *GTF3C1* | c.2177C>G | 0.01 | 0.535 | 22.9 |
| UIG009 | *TMEM88* | c.329A>C | 0.01 | 0.998 | 28.3 |
| UIG009 | *UNC45B* | c.1328A>G | 0 | 0.999 | 32 |
| UIG009 | *KRTAP4-8* | c.314G>T | 0 | 0.697 | 23.2 |
| UIG009 | *SBSN* | c.523C>G | 0.01 | 0.983 | 22.2 |
| UIG011 | *HNRNPCL1* | c.401C>T | 0.04 | 0.824 | 21.4 |
| UIG011 | *CGN* | c.2312A>G | 0.01 | 0.999 | 33 |
| UIG011 | *CHPF* | c.1301A>C | 0.01 | 0.998 | 25.6 |
| UIG011 | *SLC38A3* | c.1136T>C | 0 | 0.78 | 28 |
| UIG011 | *MAML3* | c.230T>C | 0 | 0.999 | 32 |
| UIG011 | *SH2B2* | c.83A>G | 0 | 0.735 | 31 |
| UIG011 | *AGO2* | c.1831A>T | 0 | 0.803 | 24.5 |
| UIG011 | *C9orf85* | c.412G>T | 0.01 | 0.471 | 24.2 |
| UIG011 | *ASTN2* | c.2135A>G | 0 | 0.986 | 32 |
| UIG011 | *SKIDA1* | c.302T>C | 0 | 0.998 | 32 |
| UIG011 | *SKIDA1* | c.299T>C | 0 | 0.991 | 31 |
| UIG011 | *CTBP2* | c.1932A>G | 0.03 | 0.666 | 22.7 |
| UIG011 | *CTBP2* | c.1928G>A | 0.01 | 0.984 | 30 |
| UIG011 | *CTBP2* | c.1902G>T | 0.04 | 0.854 | 26.5 |
| UIG011 | *CTBP2* | c.1873A>T | 0.01 | 0.862 | 30 |
| UIG011 | *MUC6* | c.4903A>C | 0.02 | 0.991 | 22.4 |
| UIG011 | *MUC6* | c.4487C>T | 0.04 | 0.54 | 22 |
| UIG011 | *PPP1R12A* | c.20A>T | 0 | 0.983 | 32 |
| UIG011 | *HAGHL* | c.437A>G | 0 | 0.998 | 33 |
| UIG011 | *WDR81* | c.4019C>T | 0 | 0.987 | 25.8 |
| UIG011 | *CHD3* | c.608C>G | 0.02 | 0.578 | 21 |
| UIG011 | *FBN3* | c.7805A>G | 0.02 | 0.932 | 25.3 |
| UIG011 | *PPP1R13L* | c.1576T>C | 0 | 0.897 | 28.1 |
| UIG011 | *PRRG1* | c.514T>A | 0 | 0.994 | 25.1 |
| UIG018 | *FAM43B* | c.356T>C | 0.02 | 0.708 | 27.8 |
| UIG018 | *CHPF* | c.1301A>C | 0.01 | 0.998 | 25.6 |
| UIG018 | *DUSP28* | c.269A>G | 0.01 | 0.97 | 32 |
| UIG018 | *SOWAHA* | c.695T>C | 0.02 | 0.795 | 24 |
| UIG018 | *TNXB* | c.2960A>G | 0 | 0.736 | 28.5 |
| UIG018 | *KMT2C* | c.1042G>A | 0.02 | 0.99 | 29 |
| UIG018 | *WNK2* | c.3983A>G | 0.02 | 0.523 | 25.6 |
| UIG018 | *SVIL* | c.4121T>G | 0 | 0.506 | 26.4 |
| UIG018 | *NANOS1* | c.167C>A | 0.04 | 0.967 | 24.8 |
| UIG018 | *CTBP2* | c.2113G>A | 0.03 | 0.68 | 26.5 |
| UIG018 | *PKP3* | c.1939A>G | 0.01 | 0.955 | 29 |
| UIG018 | *MYOD1* | c.326A>C | 0.02 | 0.999 | 31 |
| UIG018 | *TAS2R30* | c.142C>G | 0.05 | 0.519 | 21.2 |
| UIG018 | *DDX11* | c.556C>T | 0.02 | 0.533 | 22.4 |
| UIG018 | *ZC3H10* | c.1231A>G | 0.02 | 0.899 | 26.1 |
| UIG018 | *PABPC3* | c.1720G>A | 0 | 0.993 | 23.4 |
| UIG018 | *CTRB2* | c.185G>A | 0 | 0.999 | 25.7 |
| UIG018 | *MUC16* | c.40754C>T | 0.04 | 0.964 | 20.4 |
| UIG018 | *MUC16* | c.40753A>C | 0 | 0.964 | 21.9 |
| UIG020 | *KIF26B* | c.3713A>G | 0.01 | 0.996 | 29 |
| UIG020 | *TEKT4* | c.1031G>T | 0 | 1 | 24.5 |
| UIG020 | *TEKT4* | c.1051C>T | 0 | 0.999 | 22.7 |
| UIG020 | *CDC25A* | c.32G>C | 0.01 | 0.753 | 29.9 |
| UIG020 | *HLA-B* | c.589G>A | 0.02 | 0.53 | 24.1 |
| UIG020 | *ZNF318* | c.265T>C | 0.01 | 0.994 | 26.6 |
| UIG020 | *TUBB8* | c.554C>T | 0.01 | 0.981 | 21.9 |
| UIG020 | *CTBP2* | c.2810G>A | 0 | 0.731 | 24.6 |
| UIG020 | *ESRRA* | c.1055G>C | 0.03 | 0.888 | 27 |
| UIG020 | *OR11H12* | c.407A>G | 0 | 0.995 | 22.9 |
| UIG020 | *AHNAK2* | c.10373A>G | 0 | 0.786 | 21.3 |
| UIG020 | *FGF7* | c.457T>G | 0.03 | 0.669 | 25.9 |
| UIG020 | *FANCA* | c.4307T>C | 0 | 0.978 | 26.4 |
| UIG020 | *AATK* | c.3524T>C | 0 | 0.998 | 29.4 |
| UIG031 | *PDE4DIP* | c.4271T>C | 0 | 0.91 | 25.5 |
| UIG031 | *GAK* | c.521T>A | 0 | 0.998 | 28 |
| UIG031 | *DMXL1* | c.2492A>G | 0 | 0.5 | 24.4 |
| UIG031 | *ERVW-1* | c.1181G>A | 0.02 | 1 | 22.3 |
| UIG031 | *ERVW-1* | c.1171G>A | 0 | 0.999 | 22.3 |
| UIG031 | *ST7* | c.542A>G | 0 | 0.772 | 28.8 |
| UIG031 | *DERL1* | c.32T>C | 0.01 | 0.772 | 31 |
| UIG031 | *CTBP2* | c.2362C>T | 0.01 | 0.962 | 27.1 |
| UIG031 | *CTBP2* | c.1723C>G | 0.02 | 0.962 | 24.4 |
| UIG031 | *CTBP2* | c.1701C>A | 0.04 | 0.548 | 26.2 |
| UIG031 | *WDR89* | c.335G>A | 0 | 0.96 | 25.4 |
| UIG031 | *WDR89* | c.294T>A | 0 | 0.695 | 22.8 |
| UIG031 | *WDR89* | c.266G>A | 0 | 0.876 | 24.2 |
| UIG031 | *WDR89* | c.259T>C | 0 | 0.999 | 25.4 |
| UIG031 | *RBFOX1* | c.112G>T | 0 | 0.991 | 27.8 |
| UIG031 | *RBFOX1* | c.113G>T | 0.02 | 0.88 | 25.6 |
| UIG031 | *PSPN* | c.326T>C | 0 | 0.999 | 25.5 |
| UIG031 | *MUC16* | c.40654A>C | 0.04 | 0.987 | 23 |
| UIG031 | *SLC25A5* | c.548G>T | 0 | 0.996 | 27.2 |
| UIG038 | *CROCC* | c.2752C>T | 0 | 0.915 | 26.6 |
| UIG038 | *ANKRD36* | c.1681G>T | 0 | 0.999 | 20.4 |
| UIG038 | *FAM8A1* | c.1058G>A | 0.05 | 0.974 | 32 |
| UIG038 | *PRDM13* | c.1295A>G | 0 | 0.946 | 27.2 |
| UIG038 | *SOD2* | c.116A>G | 0 | 0.634 | 26.3 |
| UIG038 | *ERVW-1* | c.1171G>A | 0 | 0.999 | 22.3 |
| UIG038 | *FPGS* | c.413A>G | 0 | 0.999 | 32 |
| UIG038 | *CTBP2* | c.2362C>T | 0.01 | 0.962 | 27.1 |
| UIG038 | *CTBP2* | c.2144C>A | 0 | 1 | 27.6 |
| UIG038 | *CTBP2* | c.2143G>A | 0.02 | 0.982 | 26.6 |
| UIG038 | *CTBP2* | c.2141G>T | 0.01 | 0.975 | 26.2 |
| UIG038 | *GXYLT1* | c.794A>G | 0 | 0.986 | 31 |
| UIG038 | *SERPINA1* | c.1076A>T | 0.02 | 0.99 | 25.3 |
| UIG038 | *SERPINA1* | c.1075A>G | 0.03 | 0.767 | 25.3 |
| UIG038 | *AHNAK2* | c.13343A>G | 0.01 | 0.93 | 22.3 |
| UIG038 | *RNPS1* | c.865T>C | 0.03 | 0.91 | 26.4 |
| UIG038 | *AATK* | c.3524T>C | 0 | 0.998 | 29.4 |
| UIG038 | *POLD1* | c.2648T>C | 0 | 0.987 | 27.6 |
| UIG044 | *HRNR* | c.6794A>T | 0.02 | 0.637 | 20.9 |
| UIG044 | *TEKT4* | c.1276C>A | 0 | 1 | 22.5 |
| UIG044 | *SPEG* | c.2051A>C | 0.01 | 0.452 | 26.6 |
| UIG044 | *TNXB* | c.2960A>G | 0 | 0.736 | 28.5 |
| UIG044 | *CCN2* | c.650T>C | 0.02 | 0.872 | 29.5 |
| UIG044 | *C9orf85* | c.412G>T | 0.01 | 0.471 | 24.2 |
| UIG044 | *CTBP2* | c.2109G>C | 0.01 | 0.907 | 24.3 |
| UIG044 | *MUC6* | c.4487C>T | 0.04 | 0.54 | 22 |
| UIG044 | *MARK3* | c.530G>A | 0 | 0.992 | 28.4 |
| UIG044 | *AHNAK2* | c.11405A>G | 0.04 | 0.471 | 21.7 |
| UIG044 | *RNF215* | c.851A>G | 0.03 | 0.946 | 29.3 |
| UIG044 | *NDUFA6* | c.326A>T | 0.04 | 0.925 | 29.5 |
| UIG047 | *FAM43B* | c.356T>C | 0.02 | 0.708 | 27.8 |
| UIG047 | *TRIM71* | c.1166T>C | 0 | 0.991 | 27.3 |
| UIG047 | *ZNF197* | c.194A>G | 0 | 0.992 | 27.4 |
| UIG047 | *ZNF660-ZNF197* | c.194A>G | 0 | 0.992 | 27.4 |
| UIG047 | *APEH* | c.787T>G | 0 | 0.899 | 32 |
| UIG047 | *USP17L18* | c.307C>T | 0 | 1 | 22.7 |
| UIG047 | *MAML3* | c.230T>C | 0 | 0.999 | 32 |
| UIG047 | *FAM8A1* | c.1012C>A | 0 | 0.974 | 25.2 |
| UIG047 | *FAM8A1* | c.1022T>A | 0 | 0.993 | 29.2 |
| UIG047 | *FAM8A1* | c.1058G>A | 0.05 | 0.974 | 32 |
| UIG047 | *FAM8A1* | c.1071T>G | 0.02 | 0.567 | 23.4 |
| UIG047 | *ASTN2* | c.2135A>G | 0 | 0.986 | 32 |
| UIG047 | *PTGDR2* | c.662T>C | 0.02 | 0.895 | 24.5 |
| UIG047 | *UNC93B1* | c.1615T>G | 0 | 0.933 | 32 |
| UIG047 | *C1QTNF9B* | c.727T>A | 0 | 1 | 23.9 |
| UIG047 | *APOE* | c.497T>C | 0 | 0.694 | 24.7 |
| UIG048 | *CHPF* | c.1301A>C | 0.01 | 0.998 | 25.6 |
| UIG048 | *HLA-DRB5* | c.485G>A | 0.03 | 0.788 | 23 |
| UIG048 | *PTGDR2* | c.662T>C | 0.02 | 0.895 | 24.5 |
| UIG048 | *MUS81* | c.28A>G | 0 | 0.994 | 29.1 |
| UIG048 | *KRT18* | c.127G>T | 0 | 0.993 | 32 |
| UIG048 | *PABPC3* | c.1720G>A | 0 | 0.993 | 23.4 |
| UIG048 | *HCN2* | c.2069T>C | 0.01 | 0.652 | 28.1 |
| UIG054 | *CROCC* | c.2752C>T | 0 | 0.915 | 26.6 |
| UIG054 | *MUC20* | c.443G>T | 0.01 | 0.992 | 23.9 |
| UIG054 | *UNC93B1* | c.1615T>G | 0 | 0.933 | 32 |
| UIG054 | *KRTAP5-7* | c.103T>C | 0 | 0.919 | 20.7 |
| UIG054 | *GXYLT1* | c.659T>G | 0 | 0.579 | 28.8 |
| UIG054 | *AHNAK2* | c.9383A>G | 0 | 0.981 | 22.7 |
| UIG056 | *TUBA3D* | c.466C>T | 0 | 0.975 | 24.5 |
| UIG056 | *HLA-DRB1* | c.89G>T | 0.01 | 0.998 | 22.7 |
| UIG056 | *KMT2C* | c.1042G>A | 0.02 | 0.99 | 29 |
| UIG056 | *PDZD8* | c.56T>C | 0 | 0.48 | 27.6 |
| UIG056 | *CTBP2* | c.1955A>C | 0 | 0.904 | 32 |
| UIG056 | *PABPC3* | c.1720G>A | 0 | 0.993 | 23.4 |
| UIG056 | *DHRS4* | c.582G>T | 0 | 0.653 | 23.3 |
| UIG056 | *FKBP3* | c.38A>G | 0.01 | 0.647 | 32 |
| UIG056 | *STAT5B* | c.790A>G | 0.03 | 0.921 | 27.2 |
| UIG057 | *FAM8A1* | c.1012C>A | 0 | 0.974 | 25.2 |
| UIG057 | *FAM8A1* | c.1022T>A | 0 | 0.993 | 29.2 |
| UIG057 | *ZNF710* | c.1181T>C | 0 | 0.997 | 28.4 |
| UIG057 | *NTN3* | c.1637T>C | 0 | 0.999 | 25.9 |
| UIG057 | *SLC25A5* | c.548G>T | 0 | 0.996 | 27.2 |
| UIG057 | *SLC25A5* | c.597G>T | 0 | 0.689 | 24.8 |
| UIG058 | *ZNF717* | c.1546T>G | 0 | 0.999 | 22.8 |
| UIG058 | *FAM8A1* | c.1130C>T | 0.02 | 0.998 | 27.1 |
| UIG058 | *HLA-DRB5* | c.485G>A | 0.03 | 0.788 | 23 |
| UIG058 | *PLEC* | c.5645A>T | 0.04 | 0.996 | 27.6 |
| UIG058 | *SNCG* | c.5A>G | 0 | 0.999 | 30 |
| UIG058 | *CTBP2* | c.1902G>T | 0.04 | 0.854 | 26.5 |
| UIG058 | *CTBP2* | c.1873A>T | 0.01 | 0.862 | 30 |
| UIG058 | *FOLH1* | c.568C>T | 0 | 0.673 | 24.7 |
| UIG058 | *NLRP1* | c.1274A>G | 0.02 | 0.984 | 22.5 |
| UIG058 | *S1PR4* | c.71T>C | 0 | 0.916 | 25.9 |
| UIG058 | *POTEH* | c.931A>T | 0.01 | 0.801 | 22.7 |
| UIG063 | *DMRTB1* | c.167T>C | 0 | 0.998 | 28 |
| UIG063 | *MAGI3* | c.3177T>G | 0.01 | 0.989 | 23.5 |
| UIG063 | *NHLH2* | c.209G>C | 0.03 | 0.931 | 29.9 |
| UIG063 | *LCE1F* | c.232A>G | 0 | 0.953 | 24.7 |
| UIG063 | *ANKRD36* | c.1681G>T | 0 | 0.999 | 20.4 |
| UIG063 | *CRYBG3* | c.3487T>C | 0 | 0.996 | 23.7 |
| UIG063 | *FOXD4L5* | c.506T>C | 0 | 0.573 | 26.4 |
| UIG063 | *LRRC26* | c.497T>C | 0 | 1 | 25.9 |
| UIG063 | *SVIL* | c.4121T>G | 0 | 0.506 | 26.4 |
| UIG063 | *AGAP9* | c.1358A>T | 0 | 0.993 | 21.2 |
| UIG063 | *SNCG* | c.5A>G | 0 | 0.999 | 30 |
| UIG063 | *RRP12* | c.80A>C | 0 | 0.827 | 28.4 |
| UIG063 | *KCNK4* | c.812T>C | 0 | 0.883 | 32 |
| UIG063 | *GXYLT1* | c.794A>G | 0 | 0.986 | 31 |
| UIG063 | *AHNAK2* | c.9383A>G | 0 | 0.981 | 22.7 |
| UIG063 | *AHNAK2* | c.5918A>G | 0.01 | 0.979 | 20.3 |
| UIG063 | *PLIN1* | c.1520A>G | 0.03 | 0.991 | 25.4 |
| UIG063 | *ALOX15B* | c.1910T>C | 0.01 | 0.934 | 23.6 |
| UIG063 | *DESI1* | c.71T>C | 0 | 0.961 | 32 |
| UIG067 | *CHPF* | c.1301A>C | 0.01 | 0.998 | 25.6 |
| UIG067 | *ESPNL* | c.2476C>G | 0 | 0.492 | 23.1 |
| UIG067 | *GNMT* | c.124A>C | 0.02 | 0.992 | 32 |
| UIG067 | *EIF4EBP1* | c.187C>T | 0 | 0.993 | 27.3 |
| UIG067 | *EIF4EBP1* | c.194C>T | 0 | 0.997 | 28.5 |
| UIG067 | *EIF4EBP1* | c.212C>T | 0 | 0.999 | 26.5 |
| UIG067 | *DERL1* | c.32T>C | 0.01 | 0.772 | 31 |
| UIG067 | *EPPK1* | c.533G>C | 0 | 0.999 | 22.6 |
| UIG067 | *DMRT3* | c.46T>C | 0.01 | 0.979 | 23.8 |
| UIG067 | *CTBP2* | c.2113G>A | 0.03 | 0.68 | 26.5 |
| UIG067 | *JAKMIP3* | c.2126A>G | 0 | 0.864 | 33 |
| UIG067 | *TAS2R30* | c.142C>G | 0.05 | 0.519 | 21.2 |
| UIG067 | *ATG2B* | c.98A>G | 0 | 0.829 | 32 |
| UIG067 | *AHNAK2* | c.9878A>G | 0.01 | 0.647 | 21.5 |
| UIG067 | *PLIN1* | c.1520A>G | 0.03 | 0.991 | 25.4 |
| UIG067 | *MFAP4* | c.626T>C | 0 | 0.872 | 27.9 |
| UIG067 | *JAK3* | c.2762T>C | 0.01 | 0.999 | 25.7 |
| UIG067 | *SAT1* | c.17T>C | 0 | 0.757 | 29 |
| UIG103 | *C1orf122* | c.326A>G | 0 | 0.719 | 31 |
| UIG103 | *PRSS3* | c.202C>T | 0.05 | 0.902 | 22.8 |
| UIG103 | *CTBP2* | c.2810G>A | 0 | 0.731 | 24.6 |
| UIG103 | *HPD* | c.401C>T | 0 | 0.524 | 29.8 |
| UIG103 | *SEC23A* | c.2072A>G | 0 | 0.948 | 31 |
| UIG103 | *PPP2R5C* | c.374G>A | 0 | 0.499 | 32 |
| UIG103 | *EVI5L* | c.1907T>C | 0 | 0.905 | 32 |
| UIG103 | *NECTIN2* | c.1094T>C | 0 | 0.749 | 25.6 |
| UIG111 | *OBSL1* | c.593C>A | 0 | 0.974 | 24.3 |
| UIG111 | *FAM8A1* | c.640C>T | 0 | 0.706 | 23.4 |
| UIG111 | *FAM8A1* | c.1071T>G | 0.02 | 0.567 | 23.4 |
| UIG111 | *CTBP2* | c.2113G>A | 0.03 | 0.68 | 26.5 |
| UIG111 | *KRT18* | c.127G>T | 0 | 0.993 | 32 |
| UIG111 | *CHRNA7* | c.370G>A | 0.01 | 0.957 | 32 |
| UIG111 | *DSCAM* | c.1711G>A | 0.02 | 0.986 | 26.2 |
| UIG111 | *KLHL34* | c.47T>C | 0 | 0.988 | 26.9 |
| UIG12 | *RNF223* | c.656T>C | 0 | 0.996 | 24.4 |
| UIG12 | *NBPF14* | c.5036A>T | 0 | 0.842 | 20.5 |
| UIG12 | *PLEKHA6* | c.2521A>G | 0 | 0.942 | 27.7 |
| UIG12 | *TEKT4* | c.1031G>T | 0 | 1 | 24.5 |
| UIG12 | *TEKT4* | c.1051C>T | 0 | 0.999 | 22.7 |
| UIG12 | *ZNF717* | c.1546T>G | 0 | 0.999 | 22.8 |
| UIG12 | *PRDM8* | c.800A>G | 0.02 | 0.549 | 29.9 |
| UIG12 | *GNMT* | c.124A>C | 0.02 | 0.992 | 32 |
| UIG12 | *FOXD4L5* | c.506T>C | 0 | 0.573 | 26.4 |
| UIG12 | *C9orf85* | c.412G>T | 0.01 | 0.471 | 24.2 |
| UIG12 | *TRIM14* | c.1073A>C | 0 | 0.999 | 29.7 |
| UIG12 | *CTBP2* | c.2144C>A | 0 | 1 | 27.6 |
| UIG12 | *CTBP2* | c.2143G>A | 0.02 | 0.982 | 26.6 |
| UIG12 | *CTBP2* | c.2141G>T | 0.01 | 0.975 | 26.2 |
| UIG12 | *SEC23A* | c.2072A>G | 0 | 0.948 | 31 |
| UIG12 | *AHNAK2* | c.9383A>G | 0 | 0.981 | 22.7 |
| UIG12 | *TXNDC11* | c.215T>C | 0 | 0.892 | 26.8 |
| UIG12 | *MALT1* | c.122T>C | 0 | 0.552 | 27.3 |
| UIG12 | *S1PR4* | c.71T>C | 0 | 0.916 | 25.9 |
| UIG12 | *ZSCAN5A* | c.325G>A | 0 | 0.999 | 20.2 |
| UIG12 | *KLHDC7B* | c.2768T>G | 0.02 | 0.493 | 21.2 |
| UIG12 | *TCEAL2* | c.572T>G | 0 | 0.954 | 22.8 |
| UIG128 | *TEKT4* | c.1157C>G | 0 | 0.919 | 22.6 |
| UIG128 | *PID1* | c.20A>G | 0 | 0.607 | 32 |
| UIG128 | *USP17L17* | c.13T>C | 0.02 | 0.896 | 22.4 |
| UIG128 | *FRG1* | c.457A>G | 0 | 0.536 | 24.2 |
| UIG128 | *DERL1* | c.32T>C | 0.01 | 0.772 | 31 |
| UIG128 | *SVIL* | c.4121T>G | 0 | 0.506 | 26.4 |
| UIG128 | *ASAH2B* | c.23G>A | 0 | 1 | 22.3 |
| UIG128 | *CTBP2* | c.1955A>C | 0 | 0.904 | 32 |
| UIG128 | *CTBP2* | c.1847C>T | 0 | 0.921 | 26.2 |
| UIG128 | *CTBP2* | c.1814C>T | 0.01 | 0.957 | 32 |
| UIG128 | *CTBP2* | c.1705C>A | 0 | 0.902 | 27.3 |
| UIG128 | *CTBP2* | c.1703G>T | 0.02 | 0.837 | 26 |
| UIG128 | *CTBP2* | c.1701C>A | 0.04 | 0.548 | 26.2 |
| UIG128 | *UNC93B1* | c.1615T>G | 0 | 0.933 | 32 |
| UIG128 | *DYNLL1* | c.74C>T | 0.03 | 0.511 | 29.1 |
| UIG128 | *IRS2* | c.2665A>G | 0 | 0.942 | 23.1 |
| UIG128 | *GOLGA8G* | c.760T>G | 0 | 0.732 | 22.6 |
| UIG128 | *C2CD4B* | c.809T>C | 0 | 0.971 | 27.4 |
| UIG128 | *MUC16* | c.40707G>C | 0.03 | 0.988 | 20.2 |
| UIG128 | *MUC16* | c.40654A>C | 0.04 | 0.987 | 23 |
| UIG128 | *ZNF98* | c.1139G>A | 0 | 1 | 22.1 |
| UIG128 | *SLC25A5* | c.597G>T | 0 | 0.689 | 24.8 |
| UIG14 | *GLI2* | c.2689C>T | 0.03 | 0.989 | 27.4 |
| UIG14 | *MFSD10* | c.806T>C | 0.01 | 0.959 | 28 |
| UIG14 | *FOXD4* | c.494T>C | 0 | 0.977 | 27.5 |
| UIG14 | *IL15RA* | c.68T>C | 0.01 | 0.991 | 23.8 |
| UIG14 | *PTGDR2* | c.662T>C | 0.02 | 0.895 | 24.5 |
| UIG14 | *RELA* | c.836A>G | 0 | 0.984 | 29.7 |
| UIG14 | *SPTBN2* | c.1961A>G | 0.01 | 0.85 | 26 |
| UIG14 | *SPTBN2* | c.1955A>G | 0 | 0.999 | 26.9 |
| UIG14 | *ZNF710* | c.1181T>C | 0 | 0.997 | 28.4 |
| UIG14 | *TUBB8B* | c.137G>C | 0 | 0.99 | 22.2 |
| UIG14 | *CC2D1A* | c.2707C>T | 0.01 | 0.908 | 23.2 |
| UIG14 | *RYR1* | c.7597G>C | 0.01 | 0.69 | 27.3 |
| UIG14 | *LRFN1* | c.215T>C | 0 | 1 | 28.7 |
| UIG141 | *TEKT4* | c.1031G>T | 0 | 1 | 24.5 |
| UIG141 | *TEKT4* | c.1051C>T | 0 | 0.999 | 22.7 |
| UIG141 | *TRIM71* | c.1166T>C | 0 | 0.991 | 27.3 |
| UIG141 | *USP17L17* | c.2T>G | 0 | 0.974 | 22.3 |
| UIG141 | *ESRRA* | c.1055G>C | 0.03 | 0.888 | 27 |
| UIG141 | *TMEM191B* | c.358C>G | 0 | 0.744 | 23.8 |
| UIG141 | *SLC25A5* | c.548G>T | 0 | 0.996 | 27.2 |
| UIG144 | *PLXND1* | c.230A>C | 0 | 0.997 | 28.6 |
| UIG144 | *ALG1L2* | c.569A>C | 0.04 | 0.951 | 23.6 |
| UIG144 | *HLA-B* | c.623C>A | 0.01 | 0.637 | 23.1 |
| UIG144 | *ERVW-1* | c.1181G>A | 0.02 | 1 | 22.3 |
| UIG144 | *ERVW-1* | c.1171G>A | 0 | 0.999 | 22.3 |
| UIG144 | *FOXD4L4* | c.209C>A | 0 | 0.99 | 20.8 |
| UIG144 | *HHIPL1* | c.1348C>T | 0.01 | 0.93 | 24.5 |
| UIG144 | *MMP11* | c.1A>G | 0 | 0.678 | 26.2 |
| UIG144 | *VCX3A* | c.556G>A | 0 | 0.729 | 25 |
| UIG20 | *CDK11B* | c.611A>G | 0 | 0.715 | 28.1 |
| UIG20 | *ASAP3* | c.113C>G | 0.03 | 0.758 | 25.3 |
| UIG20 | *MAGI3* | c.3177T>G | 0.01 | 0.989 | 23.5 |
| UIG20 | *SCAMP3* | c.994G>C | 0 | 0.916 | 26.4 |
| UIG20 | *TEKT4* | c.1210A>G | 0.04 | 0.77 | 20.3 |
| UIG20 | *IL17RE* | c.1838A>C | 0 | 0.966 | 31 |
| UIG20 | *ERVW-1* | c.1181G>A | 0.02 | 1 | 22.3 |
| UIG20 | *VEGFB* | c.376C>T | 0.02 | 0.656 | 24.6 |
| UIG20 | *ESRRA* | c.1055G>C | 0.03 | 0.888 | 27 |
| UIG20 | *KRTAP5-7* | c.103T>C | 0 | 0.919 | 20.7 |
| UIG20 | *OR11H12* | c.407A>G | 0 | 0.995 | 22.9 |
| UIG20 | *BCL2L2* | c.281G>C | 0 | 0.983 | 28.3 |
| UIG20 | *RBFOX1* | c.112G>T | 0 | 0.991 | 27.8 |
| UIG20 | *RBFOX1* | c.113G>T | 0.02 | 0.88 | 25.6 |
| UIG20 | *AATK* | c.3524T>C | 0 | 0.998 | 29.4 |
| UIG20 | *MUC16* | c.40766A>G | 0.03 | 0.968 | 21.9 |
| UIG20 | *MUC16* | c.40754C>T | 0.04 | 0.964 | 20.4 |
| UIG20 | *MUC16* | c.40753A>C | 0 | 0.964 | 21.9 |
| UIG27 | *NKX1-1* | c.1160T>C | 0.04 | 0.991 | 24.6 |
| UIG27 | *PROB1* | c.1862T>A | 0 | 0.912 | 31 |
| UIG27 | *RREB1* | c.3556G>C | 0 | 0.962 | 28 |
| UIG27 | *ERVW-1* | c.1181G>A | 0.02 | 1 | 22.3 |
| UIG27 | *ERVW-1* | c.1171G>A | 0 | 0.999 | 22.3 |
| UIG27 | *NRF1* | c.1231G>C | 0.03 | 0.987 | 25.8 |
| UIG27 | *KRT83* | c.1103A>G | 0 | 0.941 | 24.9 |
| UIG27 | *AHNAK2* | c.9878A>G | 0.01 | 0.647 | 21.5 |
| UIG27 | *OR4M2* | c.604A>T | 0.01 | 0.981 | 24.7 |
| UIG27 | *NOMO2* | c.1396G>A | 0.03 | 0.896 | 28.8 |
| UIG27 | *MUC16* | c.40835C>T | 0.04 | 0.964 | 21 |
| UIG27 | *MUC16* | c.40707G>C | 0.03 | 0.988 | 20.2 |
| UIG27 | *MUC16* | c.40654A>C | 0.04 | 0.987 | 23 |
| UIG27 | *GSK3A* | c.397G>A | 0.01 | 0.872 | 26.4 |
| UIG28 | *C2CD4D* | c.725A>G | 0 | 0.966 | 32 |
| UIG28 | *C2CD4D* | c.718A>G | 0.02 | 0.587 | 24.9 |
| UIG28 | *SPTA1* | c.6380T>G | 0 | 0.999 | 29.2 |
| UIG28 | *C2orf81* | c.125T>C | 0 | 1 | 26 |
| UIG28 | *HES6* | c.281A>G | 0 | 0.997 | 33 |
| UIG28 | *ALG1L2* | c.569A>C | 0.04 | 0.951 | 23.6 |
| UIG28 | *MAML3* | c.230T>C | 0 | 0.999 | 32 |
| UIG28 | *NRF1* | c.1231G>C | 0.03 | 0.987 | 25.8 |
| UIG28 | *PLEC* | c.11590T>G | 0.04 | 0.478 | 23 |
| UIG28 | *PRSS3* | c.202C>T | 0.05 | 0.902 | 22.8 |
| UIG28 | *ZSWIM8* | c.5195T>C | 0 | 0.888 | 24.6 |
| UIG28 | *ZNF592* | c.1739T>C | 0 | 0.983 | 26.3 |
| UIG28 | *TPSG1* | c.647A>G | 0 | 0.972 | 23.3 |
| UIG28 | *EEF2KMT* | c.50A>G | 0.04 | 0.448 | 27.1 |
| UIG28 | *TBC1D3B* | c.700G>T | 0.01 | 0.999 | 21.3 |
| UIG28 | *TNFRSF11A* | c.1691A>G | 0.02 | 0.601 | 24.4 |
| UIG28 | *PRR12* | c.734T>C | 0 | 0.996 | 27.8 |
| UIG28 | *LAMA5* | c.5369T>C | 0 | 1 | 31 |
| UIG9 | *FAM8A1* | c.1012C>A | 0 | 0.974 | 25.2 |
| UIG9 | *FAM8A1* | c.1022T>A | 0 | 0.993 | 29.2 |
| UIG9 | *FAM8A1* | c.1058G>A | 0.05 | 0.974 | 32 |
| UIG9 | *HLA-DRB5* | c.17T>C | 0.02 | 0.649 | 22.6 |
| UIG9 | *DERL1* | c.32T>C | 0.01 | 0.772 | 31 |
| UIG9 | *PLEC* | c.5639T>A | 0.01 | 0.997 | 28.2 |
| UIG9 | *CTBP2* | c.2165C>T | 0.01 | 0.94 | 27 |
| UIG9 | *ZNF705A* | c.139G>A | 0.01 | 0.998 | 25.3 |
| UIG9 | *AHNAK2* | c.11405A>G | 0.04 | 0.471 | 21.7 |
| UIG9 | *AHNAK2* | c.9383A>G | 0 | 0.981 | 22.7 |
| UIG9 | *RBFOX1* | c.9C>G | 0.03 | 0.983 | 24 |
| UIG9 | *MUC16* | c.40707G>C | 0.03 | 0.988 | 20.2 |
| UIG9 | *MUC16* | c.40654A>C | 0.04 | 0.987 | 23 |
