# Supplementary material for: Integrating genome and RNA sequencing to enhance diagnostic precision in cerebral palsy
Source: BMC Pediatr. 2026 Apr 14;26:484. doi: 10.1186/s12887-026-06861-z (PMC13202834; doi:10.1186/s12887-026-06861-z)
Supplement: Supplementary file 3 — Supplementary Material 3. [file 12887_2026_6861_MOESM3_ESM.docx]

**Supplementary Table S3.** **List of aberrant alternative splicing events.**

| **SampleID** | **loglr** | **df** | ***P* adjust** | **intron_ID** | **logef** | **deltapsi** | **Symbol** |
| --- | --- | --- | --- | --- | --- | --- | --- |
| UIG009 | 32.83 | 20 | 0.01 | chrX:153285049:153285128 | -0.16 | 0.07 | *MECP2* |
| UIG009 | 32.83 | 20 | 0.01 | chrX:153285049:153286018 | 0.79 | 0.00 | *MECP2* |
| UIG009 | 32.83 | 20 | 0.01 | chrX:153285049:153357642 | 4.35 | 0.08 | *MECP2* |
| UIG009 | 32.83 | 20 | 0.01 | chrX:153285049:153363061 | 3.21 | 0.02 | *MECP2* |
| UIG009 | 32.83 | 20 | 0.01 | chrX:153298008:153298890 | 0.81 | 0.00 | *MECP2* |
| UIG009 | 32.83 | 20 | 0.01 | chrX:153298008:153305243 | -0.68 | 0.00 | *MECP2* |
| UIG009 | 32.83 | 20 | 0.01 | chrX:153298008:153313968 | -0.05 | 0.00 | *MECP2* |
| UIG009 | 32.83 | 20 | 0.01 | chrX:153298008:153322392 | 1.13 | 0.01 | *MECP2* |
| UIG009 | 32.83 | 20 | 0.01 | chrX:153298008:153357642 | -0.85 | -0.11 | *MECP2* |
| UIG009 | 32.83 | 20 | 0.01 | chrX:153298008:153363061 | -1.32 | -0.04 | *MECP2* |
| UIG009 | 32.83 | 20 | 0.01 | chrX:153305354:153357642 | -1.27 | -0.01 | *MECP2* |
| UIG009 | 32.83 | 20 | 0.01 | chrX:153305354:153363061 | -1.29 | 0.00 | *MECP2* |
| UIG009 | 32.83 | 20 | 0.01 | chrX:153313730:153313968 | 0.89 | 0.00 | *MECP2* |
| UIG009 | 32.83 | 20 | 0.01 | chrX:153314111:153316925 | -1.30 | 0.00 | *MECP2* |
| UIG009 | 32.83 | 20 | 0.01 | chrX:153314111:153357642 | -1.29 | 0.00 | *MECP2* |
| UIG009 | 32.83 | 20 | 0.01 | chrX:153317033:153322392 | -1.28 | 0.00 | *MECP2* |
| UIG009 | 32.83 | 20 | 0.01 | chrX:153317033:153357642 | 1.00 | 0.01 | *MECP2* |
| UIG009 | 32.83 | 20 | 0.01 | chrX:153323973:153357642 | -1.29 | 0.00 | *MECP2* |
| UIG009 | 32.83 | 20 | 0.01 | chrX:153356639:153357642 | -1.28 | 0.00 | *MECP2* |
| UIG009 | 32.83 | 20 | 0.01 | chrX:153357765:153359837 | 0.41 | 0.00 | *MECP2* |
| UIG009 | 32.83 | 20 | 0.01 | chrX:153357765:153363061 | -0.52 | -0.04 | *MECP2* |
| UIG009 | 32.83 | 20 | 0.01 | chrX:153285049:153285128 | -0.16 | 0.07 | *MECP2* |
| UIG009 | 32.83 | 20 | 0.01 | chrX:153285049:153286018 | 0.79 | 0.00 | *MECP2* |
| UIG009 | 32.83 | 20 | 0.01 | chrX:153285049:153357642 | 4.35 | 0.08 | *MECP2* |
| UIG009 | 32.83 | 20 | 0.01 | chrX:153285049:153363061 | 3.21 | 0.02 | *MECP2* |
| UIG009 | 32.83 | 20 | 0.01 | chrX:153298008:153298890 | 0.81 | 0.00 | *MECP2* |
| UIG009 | 32.83 | 20 | 0.01 | chrX:153298008:153305243 | -0.68 | 0.00 | *MECP2* |
| UIG009 | 32.83 | 20 | 0.01 | chrX:153298008:153313968 | -0.05 | 0.00 | *MECP2* |
| UIG009 | 32.83 | 20 | 0.01 | chrX:153298008:153322392 | 1.13 | 0.01 | *MECP2* |
| UIG009 | 32.83 | 20 | 0.01 | chrX:153298008:153357642 | -0.85 | -0.11 | *MECP2* |
| UIG009 | 32.83 | 20 | 0.01 | chrX:153298008:153363061 | -1.32 | -0.04 | *MECP2* |
| UIG009 | 32.83 | 20 | 0.01 | chrX:153305354:153357642 | -1.27 | -0.01 | *MECP2* |
| UIG009 | 32.83 | 20 | 0.01 | chrX:153305354:153363061 | -1.29 | 0.00 | *MECP2* |
| UIG009 | 32.83 | 20 | 0.01 | chrX:153313730:153313968 | 0.89 | 0.00 | *MECP2* |
| UIG009 | 32.83 | 20 | 0.01 | chrX:153314111:153316925 | -1.30 | 0.00 | *MECP2* |
| UIG009 | 32.83 | 20 | 0.01 | chrX:153314111:153357642 | -1.29 | 0.00 | *MECP2* |
| UIG009 | 32.83 | 20 | 0.01 | chrX:153317033:153322392 | -1.28 | 0.00 | *MECP2* |
| UIG009 | 32.83 | 20 | 0.01 | chrX:153317033:153357642 | 1.00 | 0.01 | *MECP2* |
| UIG009 | 32.83 | 20 | 0.01 | chrX:153323973:153357642 | -1.29 | 0.00 | *MECP2* |
| UIG009 | 32.83 | 20 | 0.01 | chrX:153356639:153357642 | -1.28 | 0.00 | *MECP2* |
| UIG009 | 32.83 | 20 | 0.01 | chrX:153357765:153359837 | 0.41 | 0.00 | *MECP2* |
| UIG009 | 32.83 | 20 | 0.01 | chrX:153357765:153363061 | -0.52 | -0.04 | *MECP2* |
| UIG044 | 22.48 | 11 | 0.02 | chr17:19566812:19568261 | 2.04 | -0.08 | *ALDH3A2* |
| UIG044 | 22.48 | 11 | 0.02 | chr17:19566812:19575034 | 3.50 | 0.01 | *ALDH3A2* |
| UIG044 | 22.48 | 11 | 0.02 | chr17:19568360:19575034 | 2.46 | 0.08 | *ALDH3A2* |
| UIG044 | 22.48 | 11 | 0.02 | chr17:19568360:19578871 | -4.45 | 0.00 | *ALDH3A2* |
| UIG044 | 22.48 | 11 | 0.02 | chr17:19575269:19576464 | 2.56 | 0.01 | *ALDH3A2* |
| UIG044 | 22.48 | 11 | 0.02 | chr17:19575269:19576513 | -4.45 | 0.00 | *ALDH3A2* |
| UIG044 | 22.48 | 11 | 0.02 | chr17:19575269:19578809 | -4.46 | -0.13 | *ALDH3A2* |
| UIG044 | 22.48 | 11 | 0.02 | chr17:19575269:19578871 | 4.25 | 0.10 | *ALDH3A2* |
| UIG044 | 22.48 | 11 | 0.02 | chr17:19575269:19588588 | 3.66 | 0.03 | *ALDH3A2* |
| UIG044 | 22.48 | 11 | 0.02 | chr17:19576588:19576717 | -4.44 | 0.00 | *ALDH3A2* |
| UIG044 | 22.48 | 11 | 0.02 | chr17:19576588:19578809 | -4.45 | -0.02 | *ALDH3A2* |
| UIG044 | 22.48 | 11 | 0.02 | chr17:19576588:19578871 | 3.78 | 0.01 | *ALDH3A2* |
| UIG044 | 22.48 | 11 | 0.02 | chr17:19566812:19568261 | 2.04 | -0.08 | *ALDH3A2* |
| UIG044 | 22.48 | 11 | 0.02 | chr17:19566812:19575034 | 3.50 | 0.01 | *ALDH3A2* |
| UIG044 | 22.48 | 11 | 0.02 | chr17:19568360:19575034 | 2.46 | 0.08 | *ALDH3A2* |
| UIG044 | 22.48 | 11 | 0.02 | chr17:19568360:19578871 | -4.45 | 0.00 | *ALDH3A2* |
| UIG044 | 22.48 | 11 | 0.02 | chr17:19575269:19576464 | 2.56 | 0.01 | *ALDH3A2* |
| UIG044 | 22.48 | 11 | 0.02 | chr17:19575269:19576513 | -4.45 | 0.00 | *ALDH3A2* |
| UIG044 | 22.48 | 11 | 0.02 | chr17:19575269:19578809 | -4.46 | -0.13 | *ALDH3A2* |
| UIG044 | 22.48 | 11 | 0.02 | chr17:19575269:19578871 | 4.25 | 0.10 | *ALDH3A2* |
| UIG044 | 22.48 | 11 | 0.02 | chr17:19575269:19588588 | 3.66 | 0.03 | *ALDH3A2* |
| UIG044 | 22.48 | 11 | 0.02 | chr17:19576588:19576717 | -4.44 | 0.00 | *ALDH3A2* |
| UIG044 | 22.48 | 11 | 0.02 | chr17:19576588:19578809 | -4.45 | -0.02 | *ALDH3A2* |
| UIG044 | 22.48 | 11 | 0.02 | chr17:19576588:19578871 | 3.78 | 0.01 | *ALDH3A2* |
| UIG044 | 22.48 | 11 | 0.02 | chr17:19566812:19568261 | 2.04 | -0.08 | *ALDH3A2* |
| UIG044 | 22.48 | 11 | 0.02 | chr17:19566812:19575034 | 3.50 | 0.01 | *ALDH3A2* |
| UIG044 | 22.48 | 11 | 0.02 | chr17:19568360:19575034 | 2.46 | 0.08 | *ALDH3A2* |
| UIG044 | 22.48 | 11 | 0.02 | chr17:19568360:19578871 | -4.45 | 0.00 | *ALDH3A2* |
| UIG044 | 22.48 | 11 | 0.02 | chr17:19575269:19576464 | 2.56 | 0.01 | *ALDH3A2* |
| UIG044 | 22.48 | 11 | 0.02 | chr17:19575269:19576513 | -4.45 | 0.00 | *ALDH3A2* |
| UIG044 | 22.48 | 11 | 0.02 | chr17:19575269:19578809 | -4.46 | -0.13 | *ALDH3A2* |
| UIG044 | 22.48 | 11 | 0.02 | chr17:19575269:19578871 | 4.25 | 0.10 | *ALDH3A2* |
| UIG044 | 22.48 | 11 | 0.02 | chr17:19575269:19588588 | 3.66 | 0.03 | *ALDH3A2* |
| UIG044 | 22.48 | 11 | 0.02 | chr17:19576588:19576717 | -4.44 | 0.00 | *ALDH3A2* |
| UIG044 | 22.48 | 11 | 0.02 | chr17:19576588:19578809 | -4.45 | -0.02 | *ALDH3A2* |
| UIG044 | 22.48 | 11 | 0.02 | chr17:19576588:19578871 | 3.78 | 0.01 | *ALDH3A2* |
| UIG044 | 16.70 | 4 | 0.01 | chr19:54697173:54705028 | 0.39 | 0.00 | *TSEN34* |
| UIG044 | 16.70 | 4 | 0.01 | chr19:54704756:54705028 | 2.27 | -0.09 | *TSEN34* |
| UIG044 | 16.70 | 4 | 0.01 | chr19:54704756:54705034 | 1.48 | -0.01 | *TSEN34* |
| UIG044 | 16.70 | 4 | 0.01 | chr19:54704761:54705028 | -8.54 | -0.04 | *TSEN34* |
| UIG044 | 16.70 | 4 | 0.01 | chr19:54704829:54705028 | 4.39 | 0.14 | *TSEN34* |
| UIG048 | 12.53 | 1 | 0.00 | chr19:10274036:10277268 | 2.02 | 0.58 | *DNMT1* |
| UIG048 | 12.53 | 1 | 0.00 | chr19:10274036:10277274 | -2.02 | -0.58 | *DNMT1* |
| UIG048 | 12.53 | 1 | 0.00 | chr19:10274036:10277268 | 2.02 | 0.58 | *DNMT1* |
| UIG048 | 12.53 | 1 | 0.00 | chr19:10274036:10277274 | -2.02 | -0.58 | *DNMT1* |
| UIG048 | 18.61 | 9 | 0.03 | chr13:111315865:111316018 | -1.35 | 0.00 | *CARS2* |
| UIG048 | 18.61 | 9 | 0.03 | chr13:111315865:111316157 | 2.11 | 0.00 | *CARS2* |
| UIG048 | 18.61 | 9 | 0.03 | chr13:111315865:111317875 | 2.72 | 0.38 | *CARS2* |
| UIG048 | 18.61 | 9 | 0.03 | chr13:111315865:111319118 | -1.38 | -0.01 | *CARS2* |
| UIG048 | 18.61 | 9 | 0.03 | chr13:111315865:111319164 | -1.35 | 0.00 | *CARS2* |
| UIG048 | 18.61 | 9 | 0.03 | chr13:111315865:111319687 | 0.35 | -0.34 | *CARS2* |
| UIG048 | 18.61 | 9 | 0.03 | chr13:111316393:111317875 | 2.94 | 0.02 | *CARS2* |
| UIG048 | 18.61 | 9 | 0.03 | chr13:111316393:111319118 | -1.35 | 0.00 | *CARS2* |
| UIG048 | 18.61 | 9 | 0.03 | chr13:111316393:111319687 | -1.35 | 0.00 | *CARS2* |
| UIG048 | 18.61 | 9 | 0.03 | chr13:111319275:111319687 | -1.33 | -0.05 | *CARS2* |
| UIG048 | 12.53 | 1 | 0.00 | chr19:10274036:10277268 | 2.02 | 0.58 | *DNMT1* |
| UIG048 | 12.53 | 1 | 0.00 | chr19:10274036:10277274 | -2.02 | -0.58 | *DNMT1* |
| UIG054 | 22.69 | 9 | 0.01 | chr14:76330205:76349028 | -0.35 | -0.01 | *IFT43* |
| UIG054 | 22.69 | 9 | 0.01 | chr14:76330205:76368485 | -1.42 | 0.00 | *IFT43* |
| UIG054 | 22.69 | 9 | 0.01 | chr14:76349245:76368485 | -0.34 | -0.02 | *IFT43* |
| UIG054 | 22.69 | 9 | 0.01 | chr14:76349245:76420767 | -0.54 | 0.00 | *IFT43* |
| UIG054 | 22.69 | 9 | 0.01 | chr14:76368567:76372898 | 1.74 | 0.21 | *IFT43* |
| UIG054 | 22.69 | 9 | 0.01 | chr14:76368567:76408033 | -0.26 | 0.00 | *IFT43* |
| UIG054 | 22.69 | 9 | 0.01 | chr14:76368567:76420767 | -1.47 | -0.27 | *IFT43* |
| UIG054 | 22.69 | 9 | 0.01 | chr14:76373020:76420767 | 1.30 | 0.06 | *IFT43* |
| UIG054 | 22.69 | 9 | 0.01 | chr14:76373026:76420767 | 1.73 | 0.04 | *IFT43* |
| UIG054 | 22.69 | 9 | 0.01 | chr14:76408123:76420767 | -0.40 | -0.01 | *IFT43* |
| UIG056 | 19.07 | 8 | 0.04 | chr22:32871111:32874968 | 1.06 | 0.08 | *FBXO7* |
| UIG056 | 19.07 | 8 | 0.04 | chr22:32871111:32875120 | 0.84 | 0.02 | *FBXO7* |
| UIG056 | 19.07 | 8 | 0.04 | chr22:32871385:32874968 | -0.96 | -0.16 | *FBXO7* |
| UIG056 | 19.07 | 8 | 0.04 | chr22:32871385:32875005 | -0.95 | 0.00 | *FBXO7* |
| UIG056 | 19.07 | 8 | 0.04 | chr22:32871385:32875120 | 0.82 | 0.02 | *FBXO7* |
| UIG056 | 19.07 | 8 | 0.04 | chr22:32871385:32879884 | -0.21 | 0.00 | *FBXO7* |
| UIG056 | 19.07 | 8 | 0.04 | chr22:32871385:32881055 | -0.97 | 0.00 | *FBXO7* |
| UIG056 | 19.07 | 8 | 0.04 | chr22:32875262:32879884 | 0.20 | 0.03 | *FBXO7* |
| UIG056 | 19.07 | 8 | 0.04 | chr22:32880111:32881055 | 0.17 | 0.02 | *FBXO7* |
| UIG057 | 58.99 | 11 | 0.00 | chr14:94849578:94849759 | -0.05 | 0.00 | *SERPINA1* |
| UIG057 | 58.99 | 11 | 0.00 | chr14:94849578:94854897 | -0.14 | -0.19 | *SERPINA1* |
| UIG057 | 58.99 | 11 | 0.00 | chr14:94849578:94856757 | -0.08 | 0.00 | *SERPINA1* |
| UIG057 | 58.99 | 11 | 0.00 | chr14:94849578:94856794 | 1.36 | 0.35 | *SERPINA1* |
| UIG057 | 58.99 | 11 | 0.00 | chr14:94854997:94855138 | -0.14 | -0.01 | *SERPINA1* |
| UIG057 | 58.99 | 11 | 0.00 | chr14:94854997:94855156 | -0.14 | -0.02 | *SERPINA1* |
| UIG057 | 58.99 | 11 | 0.00 | chr14:94854997:94856794 | -0.14 | -0.02 | *SERPINA1* |
| UIG057 | 58.99 | 11 | 0.00 | chr14:94855000:94855138 | -0.14 | -0.02 | *SERPINA1* |
| UIG057 | 58.99 | 11 | 0.00 | chr14:94855000:94855156 | -0.14 | -0.03 | *SERPINA1* |
| UIG057 | 58.99 | 11 | 0.00 | chr14:94855000:94856794 | -0.14 | -0.03 | *SERPINA1* |
| UIG057 | 58.99 | 11 | 0.00 | chr14:94855347:94856186 | -0.14 | 0.00 | *SERPINA1* |
| UIG057 | 58.99 | 11 | 0.00 | chr14:94855347:94856794 | -0.14 | -0.03 | *SERPINA1* |
| UIG067 | 34.39 | 2 | 0.00 | chr15:62146757:62147070 | -7.34 | -0.28 | *VPS13C* |
| UIG067 | 34.39 | 2 | 0.00 | chr15:62146757:62148485 | 14.68 | 0.51 | *VPS13C* |
| UIG067 | 34.39 | 2 | 0.00 | chr15:62147153:62148485 | -7.34 | -0.24 | *VPS13C* |
| UIG141 | 21.35 | 7 | 0.00 | chr1:161180500:161182141 | -0.94 | 0.09 | *NDUFS2* |
| UIG141 | 21.35 | 7 | 0.00 | chr1:161180500:161183170 | -1.44 | 0.00 | *NDUFS2* |
| UIG141 | 21.35 | 7 | 0.00 | chr1:161182270:161182382 | -0.77 | 0.00 | *NDUFS2* |
| UIG141 | 21.35 | 7 | 0.00 | chr1:161182270:161183170 | -1.42 | -0.09 | *NDUFS2* |
| UIG141 | 21.35 | 7 | 0.00 | chr1:161182270:161183439 | 5.47 | 0.04 | *NDUFS2* |
| UIG141 | 21.35 | 7 | 0.00 | chr1:161182444:161183170 | 0.19 | 0.01 | *NDUFS2* |
| UIG141 | 21.35 | 7 | 0.00 | chr1:161182972:161183170 | 0.40 | 0.01 | *NDUFS2* |
| UIG141 | 21.35 | 7 | 0.00 | chr1:161183265:161183439 | -1.47 | -0.06 | *NDUFS2* |
| UIG12 | 41.71 | 2 | 0.00 | chr10:73581764:73583645 | -3.10 | -0.03 | *PSAP* |
| UIG12 | 41.71 | 2 | 0.00 | chr10:73581764:73585594 | 6.19 | 0.05 | *PSAP* |
| UIG12 | 41.71 | 2 | 0.00 | chr10:73583653:73585594 | -3.10 | -0.02 | *PSAP* |
| UIG14 | 12.67 | 2 | 0.01 | chr16:57691403:57693307 | -4.10 | -0.05 | *ADGRG1* |
| UIG14 | 12.67 | 2 | 0.01 | chr16:57691403:57693325 | -4.10 | -0.09 | *ADGRG1* |
| UIG14 | 12.67 | 2 | 0.01 | chr16:57691403:57693429 | 8.19 | 0.14 | *ADGRG1* |
| UIG14 | 10.48 | 2 | 0.04 | chr11:108143579:108144089 | 15.13 | 0.99 | *ATM* |
| UIG14 | 10.48 | 2 | 0.04 | chr11:108143579:108150218 | -7.56 | -0.98 | *ATM* |
| UIG14 | 10.48 | 2 | 0.04 | chr11:108143579:108150228 | -7.56 | -0.01 | *ATM* |
| UIG14 | 10.56 | 2 | 0.04 | chr5:7889388:7890374 | 0.79 | 0.15 | *MTRR* |
| UIG14 | 10.56 | 2 | 0.04 | chr5:7889388:7891485 | -1.29 | -0.32 | *MTRR* |
| UIG14 | 10.56 | 2 | 0.04 | chr5:7890525:7891485 | 0.50 | 0.17 | *MTRR* |
| UIG14 | 13.29 | 4 | 0.04 | chr9:94874844:94876908 | 8.73 | 0.15 | *SPTLC1* |
| UIG14 | 13.29 | 4 | 0.04 | chr9:94874844:94877204 | -2.18 | 0.00 | *SPTLC1* |
| UIG14 | 13.29 | 4 | 0.04 | chr9:94874844:94877596 | -2.18 | -0.14 | *SPTLC1* |
| UIG14 | 13.29 | 4 | 0.04 | chr9:94874844:94879674 | -2.18 | 0.00 | *SPTLC1* |
| UIG14 | 13.29 | 4 | 0.04 | chr9:94877348:94877596 | -2.18 | 0.00 | *SPTLC1* |
| UIG14 | 11.08 | 2 | 0.03 | chr17:7804236:7804562 | -5.00 | 0.00 | *CHD3* |
| UIG14 | 11.08 | 2 | 0.03 | chr17:7804311:7804562 | -5.00 | -0.11 | *CHD3* |
| UIG14 | 11.08 | 2 | 0.03 | chr17:7804311:7804624 | 10.00 | 0.11 | *CHD3* |
| UIG14 | 13.11 | 4 | 0.04 | chr12:111951343:111953958 | 0.49 | 0.25 | *ATXN2* |
| UIG14 | 13.11 | 4 | 0.04 | chr12:111951343:111956053 | -1.12 | -0.02 | *ATXN2* |
| UIG14 | 13.11 | 4 | 0.04 | chr12:111954167:111954991 | -1.90 | 0.00 | *ATXN2* |
| UIG14 | 13.11 | 4 | 0.04 | chr12:111954167:111956053 | -0.73 | -0.28 | *ATXN2* |
| UIG14 | 13.11 | 4 | 0.04 | chr12:111954203:111956053 | 3.26 | 0.06 | *ATXN2* |
| UIG14 | 21.34 | 8 | 0.00 | chr4:83750211:83752090 | 2.29 | 0.00 | *SEC31A* |
| UIG14 | 21.34 | 8 | 0.00 | chr4:83750211:83763293 | 1.90 | 0.01 | *SEC31A* |
| UIG14 | 21.34 | 8 | 0.00 | chr4:83750211:83763338 | -2.69 | -0.01 | *SEC31A* |
| UIG14 | 21.34 | 8 | 0.00 | chr4:83750211:83765539 | 3.49 | 0.06 | *SEC31A* |
| UIG14 | 21.34 | 8 | 0.00 | chr4:83752128:83763293 | 1.45 | 0.00 | *SEC31A* |
| UIG14 | 21.34 | 8 | 0.00 | chr4:83763634:83764450 | -2.68 | 0.00 | *SEC31A* |
| UIG14 | 21.34 | 8 | 0.00 | chr4:83763634:83765539 | 1.65 | -0.05 | *SEC31A* |
| UIG14 | 21.34 | 8 | 0.00 | chr4:83763634:83765564 | -2.70 | 0.00 | *SEC31A* |
| UIG14 | 21.34 | 8 | 0.00 | chr4:83764600:83765539 | -2.70 | -0.01 | *SEC31A* |
| UIG14 | 26.40 | 17 | 0.03 | chr19:38800283:38801712 | -4.13 | 0.00 | *YIF1B* |
| UIG14 | 26.40 | 17 | 0.03 | chr19:38800283:38804534 | 3.81 | 0.00 | *YIF1B* |
| UIG14 | 26.40 | 17 | 0.03 | chr19:38800283:38806065 | 4.38 | 0.00 | *YIF1B* |
| UIG14 | 26.40 | 17 | 0.03 | chr19:38800283:38806357 | 5.00 | 0.16 | *YIF1B* |
| UIG14 | 26.40 | 17 | 0.03 | chr19:38800283:38806363 | 5.00 | 0.09 | *YIF1B* |
| UIG14 | 26.40 | 17 | 0.03 | chr19:38800283:38806387 | 4.28 | 0.00 | *YIF1B* |
| UIG14 | 26.40 | 17 | 0.03 | chr19:38800283:38806500 | 4.19 | -0.01 | *YIF1B* |
| UIG14 | 26.40 | 17 | 0.03 | chr19:38800283:38806509 | 3.96 | -0.12 | *YIF1B* |
| UIG14 | 26.40 | 17 | 0.03 | chr19:38800283:38806738 | -4.14 | -0.01 | *YIF1B* |
| UIG14 | 26.40 | 17 | 0.03 | chr19:38800283:38807720 | -4.14 | 0.00 | *YIF1B* |
| UIG14 | 26.40 | 17 | 0.03 | chr19:38800283:38807777 | -4.13 | 0.00 | *YIF1B* |
| UIG14 | 26.40 | 17 | 0.03 | chr19:38800283:38807792 | 2.38 | -0.05 | *YIF1B* |
| UIG14 | 26.40 | 17 | 0.03 | chr19:38800283:38807816 | 4.26 | 0.00 | *YIF1B* |
| UIG14 | 26.40 | 17 | 0.03 | chr19:38800283:38807886 | -4.14 | 0.00 | *YIF1B* |
| UIG14 | 26.40 | 17 | 0.03 | chr19:38800283:38807918 | -4.14 | 0.00 | *YIF1B* |
| UIG14 | 26.40 | 17 | 0.03 | chr19:38800283:38812468 | -4.15 | -0.05 | *YIF1B* |
| UIG14 | 26.40 | 17 | 0.03 | chr19:38806246:38806357 | -4.14 | 0.00 | *YIF1B* |
| UIG14 | 26.40 | 17 | 0.03 | chr19:38806839:38807792 | -4.15 | -0.01 | *YIF1B* |
| UIG28 | 10.64 | 3 | 0.03 | chrX:40440354:40448238 | -0.90 | -0.08 | *ATP6AP2* |
| UIG28 | 10.64 | 3 | 0.03 | chrX:40440354:40450486 | 0.57 | 0.02 | *ATP6AP2* |
| UIG28 | 10.64 | 3 | 0.03 | chrX:40448368:40449656 | 0.97 | 0.01 | *ATP6AP2* |
| UIG28 | 10.64 | 3 | 0.03 | chrX:40448368:40450486 | -0.64 | 0.05 | *ATP6AP2* |
| UIG28 | 23.60 | 14 | 0.01 | chr3:184032462:184033277 | -0.15 | 0.02 | *EIF4G1* |
| UIG28 | 23.60 | 14 | 0.01 | chr3:184032462:184033551 | 0.30 | 0.01 | *EIF4G1* |
| UIG28 | 23.60 | 14 | 0.01 | chr3:184032462:184035109 | 0.12 | 0.01 | *EIF4G1* |
| UIG28 | 23.60 | 14 | 0.01 | chr3:184032462:184035505 | -0.93 | 0.00 | *EIF4G1* |
| UIG28 | 23.60 | 14 | 0.01 | chr3:184032462:184038418 | 3.17 | 0.03 | *EIF4G1* |
| UIG28 | 23.60 | 14 | 0.01 | chr3:184033333:184033551 | -0.06 | 0.05 | *EIF4G1* |
| UIG28 | 23.60 | 14 | 0.01 | chr3:184033333:184035109 | -0.90 | 0.00 | *EIF4G1* |
| UIG28 | 23.60 | 14 | 0.01 | chr3:184033644:184033860 | -0.93 | 0.00 | *EIF4G1* |
| UIG28 | 23.60 | 14 | 0.01 | chr3:184033644:184033920 | -0.29 | 0.01 | *EIF4G1* |
| UIG28 | 23.60 | 14 | 0.01 | chr3:184033644:184033924 | 0.79 | 0.00 | *EIF4G1* |
| UIG28 | 23.60 | 14 | 0.01 | chr3:184033644:184035109 | -0.34 | 0.00 | *EIF4G1* |
| UIG28 | 23.60 | 14 | 0.01 | chr3:184034006:184035109 | -0.94 | -0.07 | *EIF4G1* |
| UIG28 | 23.60 | 14 | 0.01 | chr3:184035219:184035505 | 1.40 | 0.01 | *EIF4G1* |
| UIG28 | 23.60 | 14 | 0.01 | chr3:184035285:184035505 | -0.43 | 0.00 | *EIF4G1* |
| UIG28 | 23.60 | 14 | 0.01 | chr3:184037589:184038418 | -0.80 | -0.07 | *EIF4G1* |
| UIG28 | 12.66 | 2 | 0.01 | chr7:150698513:150698632 | -9.01 | -0.76 | *NOS3* |
| UIG28 | 12.66 | 2 | 0.01 | chr7:150698513:150698909 | 18.02 | 1.00 | *NOS3* |
| UIG28 | 12.66 | 2 | 0.01 | chr7:150698705:150698909 | -9.01 | -0.24 | *NOS3* |
| UIG28 | 17.09 | 3 | 0.00 | chr1:8031023:8037712 | -0.70 | -0.12 | *PARK7* |
| UIG28 | 17.09 | 3 | 0.00 | chr1:8031023:8044954 | -0.21 | 0.00 | *PARK7* |
| UIG28 | 17.09 | 3 | 0.00 | chr1:8031060:8037712 | 0.96 | 0.00 | *PARK7* |
| UIG28 | 17.09 | 3 | 0.00 | chr1:8037798:8044954 | -0.05 | 0.11 | *PARK7* |
| UIG28 | 8.40 | 2 | 0.05 | chr3:42242546:42243928 | -1.34 | 0.12 | *TRAK1* |
| UIG28 | 8.40 | 2 | 0.05 | chr3:42242546:42251259 | 3.37 | 0.10 | *TRAK1* |
| UIG28 | 8.40 | 2 | 0.05 | chr3:42244244:42251259 | -2.03 | -0.21 | *TRAK1* |
| UIG28 | 11.56 | 3 | 0.02 | chr13:38928433:38932230 | -2.87 | -0.04 | *UFM1* |
| UIG28 | 11.56 | 3 | 0.02 | chr13:38928433:38933438 | -2.53 | 0.00 | *UFM1* |
| UIG28 | 11.56 | 3 | 0.02 | chr13:38932269:38933438 | -2.87 | -0.05 | *UFM1* |
| UIG28 | 11.56 | 3 | 0.02 | chr13:38932269:38936622 | 8.28 | 0.09 | *UFM1* |
| UIG28 | 8.56 | 2 | 0.05 | chr5:60831446:60834641 | -1.34 | -0.27 | *ZSWIM6* |
| UIG28 | 8.56 | 2 | 0.05 | chr5:60831446:60835423 | 1.58 | 0.01 | *ZSWIM6* |
| UIG28 | 8.56 | 2 | 0.05 | chr5:60834798:60835423 | -0.24 | 0.26 | *ZSWIM6* |
| UIG30 | 22.61 | 6 | 0.00 | chr12:40634419:40635024 | 5.37 | 0.42 | *LRRK2* |
| UIG30 | 22.61 | 6 | 0.00 | chr12:40634419:40637352 | -0.89 | -0.14 | *LRRK2* |
| UIG30 | 22.61 | 6 | 0.00 | chr12:40634419:40643628 | -0.89 | 0.00 | *LRRK2* |
| UIG30 | 22.61 | 6 | 0.00 | chr12:40637483:40639312 | -0.89 | 0.00 | *LRRK2* |
| UIG30 | 22.61 | 6 | 0.00 | chr12:40637483:40640603 | -0.89 | 0.00 | *LRRK2* |
| UIG30 | 22.61 | 6 | 0.00 | chr12:40637483:40641692 | -0.89 | 0.00 | *LRRK2* |
| UIG30 | 22.61 | 6 | 0.00 | chr12:40637483:40643628 | -0.89 | -0.28 | *LRRK2* |
| UIG30 | 23.71 | 13 | 0.03 | chr16:15785180:15785667 | 2.20 | 0.02 | *NDE1* |
| UIG30 | 23.71 | 13 | 0.03 | chr16:15785180:15788022 | 0.59 | -0.04 | *NDE1* |
| UIG30 | 23.71 | 13 | 0.03 | chr16:15785180:15790566 | 0.56 | 0.00 | *NDE1* |
| UIG30 | 23.71 | 13 | 0.03 | chr16:15785765:15788022 | 3.30 | 0.06 | *NDE1* |
| UIG30 | 23.71 | 13 | 0.03 | chr16:15788113:15789765 | -2.91 | 0.00 | *NDE1* |
| UIG30 | 23.71 | 13 | 0.03 | chr16:15788113:15790566 | 0.63 | -0.01 | *NDE1* |
| UIG30 | 23.71 | 13 | 0.03 | chr16:15788113:15793454 | 1.83 | 0.01 | *NDE1* |
| UIG30 | 23.71 | 13 | 0.03 | chr16:15788113:15793586 | 1.17 | 0.00 | *NDE1* |
| UIG30 | 23.71 | 13 | 0.03 | chr16:15788113:15818048 | 0.44 | -0.01 | *NDE1* |
| UIG30 | 23.71 | 13 | 0.03 | chr16:15789843:15790566 | -2.92 | -0.01 | *NDE1* |
| UIG30 | 23.71 | 13 | 0.03 | chr16:15790717:15793454 | -2.92 | -0.01 | *NDE1* |
| UIG30 | 23.71 | 13 | 0.03 | chr16:15790717:15793586 | 0.64 | 0.00 | *NDE1* |
| UIG30 | 23.71 | 13 | 0.03 | chr16:15790717:15818048 | 0.32 | -0.02 | *NDE1* |
| UIG30 | 23.71 | 13 | 0.03 | chr16:15793711:15818048 | -2.92 | -0.01 | *NDE1* |
