# Supplementary material for: Integrating genome and RNA sequencing to enhance diagnostic precision in cerebral palsy
Source: BMC Pediatr. 2026 Apr 14;26:484. doi: 10.1186/s12887-026-06861-z (PMC13202834; doi:10.1186/s12887-026-06861-z)
Supplement: Supplementary file 4 — Supplementary Material 4. [file 12887_2026_6861_MOESM4_ESM.docx]

**Supplementary Table S4.**

**Summary of splicing sites in genes with aberrant alternative splicing events.**

| **Case** | **SYMBOL** | **HGVSc** | **DS_DL *** | **Inheritance** | **OMIM ID** |
| --- | --- | --- | --- | --- | --- |
| **UIG048** | DNMT1 | n.503+2C>T | 0.93 | AD | 604121, 614116 |
| **UIG057** | SERPINA1 | c.1066-4C>T | 0 | AR | 613490 |
| **UIG057** | SERPINA1 | c.1065+6A>G | 0 |  |  |
| **UIG067** | VPS13C | c.11160+1G>A | 0.95 | AR | 616840 |
| **UIG141** | NDUFS2 | c.1212G>A | 0.09 | AR | 620569, 618228 |

* DS_DL: Delta score (donor loss)
